# Germline genetic regulation of the colorectal tumor immune microenvironment

**DOI:** 10.1186/s12864-024-10295-1

**Published:** 2024-04-25

**Authors:** Stephanie L. Schmit, Ya-Yu Tsai, Joseph D. Bonner, Rebeca Sanz-Pamplona, Amit D. Joshi, Tomotaka Ugai, Sidney S. Lindsey, Marilena Melas, Kevin J. McDonnell, Gregory E. Idos, Christopher P. Walker, Chenxu Qu, W. Martin Kast, Diane M. Da Silva, Jonathan N. Glickman, Andrew T. Chan, Marios Giannakis, Jonathan A. Nowak, Hedy S. Rennert, Harlan S. Robins, Shuji Ogino, Joel K. Greenson, Victor Moreno, Gad Rennert, Stephen B. Gruber

**Affiliations:** 1 Genomic Medicine Institute, Cleveland Clinic, Cleveland, OH USA; 2 Population and Cancer Prevention Program, Case Comprehensive Cancer Center, Cleveland, OH USA; 3 Center for Precision Medicine, City of Hope National Medical Center, Duarte, CA USA; 4 Catalan Institute of Oncology (ICO), Hospitalet de Llobregat, Barcelona, Spain; 5 ONCOBELL Program, Bellvitge Biomedical Research Institute (IDIBELL), Hospitalet de Llobregat, Barcelona, Spain; 6 Consortium for Biomedical Research in Epidemiology and Public Health (CIBERESP), Barcelona, Spain; 7 Clinical and Translational Epidemiology Unit, Massachusetts General Hospital, Harvard Medical School, Boston, MA USA; 8 Department of Epidemiology, Harvard T.H. Chan School of Public Health, Harvard University, Boston, MA USA; 9 Program in MPE Molecular Pathological Epidemiology, Department of Pathology, Brigham and Women’s Hospital, Boston, MA USA; 10 Division of Gastroenterology, Massachusetts General Hospital, Harvard Medical School, Boston, MA USA; 11 Department of Pathology, Massachusetts General Hospital, Boston, MA USA; 12 Norris Comprehensive Cancer Center, University of Southern California, Los Angeles, CA USA; 13 Channing Division of Network Medicine, Brigham and Women’s Hospital, Harvard Medical School, Boston, MA USA; 14 Broad Institute of MIT and Harvard, Cambridge, MA USA; 15 Department of Immunology and Infectious Diseases, Harvard T.H. Chan School of Public Health, Harvard University, Boston, MA USA; 16 Dana-Farber Cancer Institute, Boston, MA USA; 17 B. Rappaport Faculty of Medicine, Technion and the Association for Promotion of Research in Precision Medicine (APRPM), Haifa, Israel; 18 Adaptive Biotechnologies Corp, Seattle, WA USA; 19 Tokyo Medical and Dental University (Institute of Science Tokyo), Tokyo, Japan; 20 Department of Pathology, University of Michigan, Ann Arbor, MI USA; 21 Department of Clinical Sciences, Faculty of Medicine, University of Barcelona, Barcelona, Spain

**Keywords:** Genetic polymorphisms, T cell responses, Colorectal Cancer

## Abstract

**Objective:**

To evaluate the contribution of germline genetics to regulating the briskness and diversity of T cell responses in CRC, we conducted a genome-wide association study to examine the associations between germline genetic variation and quantitative measures of T cell landscapes in 2,876 colorectal tumors from participants in the Molecular Epidemiology of Colorectal Cancer Study (MECC).

**Methods:**

Germline DNA samples were genotyped and imputed using genome-wide arrays. Tumor DNA samples were extracted from paraffin blocks, and T cell receptor clonality and abundance were quantified by immunoSEQ (Adaptive Biotechnologies, Seattle, WA). Tumor infiltrating lymphocytes per high powered field (TILs/hpf) were scored by a gastrointestinal pathologist. Regression models were used to evaluate the associations between each variant and the three T-cell features, adjusting for sex, age, genotyping platform, and global ancestry. Three independent datasets were used for replication.

**Results:**

We identified a SNP (rs4918567) near *RBM20* associated with clonality at a genome-wide significant threshold of 5 × 10^− 8^, with a consistent direction of association in both discovery and replication datasets. Expression quantitative trait (eQTL) analyses and in silico functional annotation for these loci provided insights into potential functional roles, including a statistically significant eQTL between the T allele at rs4918567 and higher expression of *ADRA2A* (*P* = 0.012) in healthy colon mucosa.

**Conclusions:**

Our study suggests that germline genetic variation is associated with the quantity and diversity of adaptive immune responses in CRC. Further studies are warranted to replicate these findings in additional samples and to investigate functional genomic mechanisms.

**Supplementary Information:**

The online version contains supplementary material available at 10.1186/s12864-024-10295-1.

## Background

 In colorectal cancer (CRC), a strong tumor infiltrating lymphocyte (TIL) response is an established prognostic indicator for better disease-specific survival and a predictive factor for response to checkpoint inhibitor immunotherapy [1–5]. However, the extent and diversity of T cell responses in the CRC tumor microenvironment are highly variable with mostly uncertain drivers. Beyond microsatellite instability (MSI) and tumor mutational burden, the factors contributing to heterogeneity in adaptive immune responses, including abundance and clonality of T cells, across tumors remain largely unexplored. Germline variation, particularly in genes important for immune cell differentiation and function, may contribute to the strength and quality of adaptive immune responses mounted against colorectal tumor cells. In turn, this variability may influence both the risk of developing cancer and subsequent clinical outcomes following a CRC diagnosis via altered immunosurveillance.

Indeed, examples of germline genetic variation modifying the diversity of constitutional immune environments and host immune responses to cancer are numerous. Single nucleotide polymorphisms (SNPs) in the Major Histocompatibility Complex locus containing *HLA* genes are highly associated with risk of Hodgkin lymphoma [6]. Further, pro-inflammatory cytokines have been strongly associated with African germline genetic ancestry proportion among African American women attributable to a Duffy-null allele [7]. Recently, three separate pan-cancer investigations utilizing The Cancer Genome Atlas (TCGA) data revealed multiple inherited genetic factors to be associated with tumor-immune compositional and functional features of cancer, including CRC [8–10]. However, each included only a limited number of CRCs and relied upon an estimation of immune infiltration from transcriptomic data. In other investigations specific to CRC, common variants in immune-related genes and pathways have been linked to CRC risk and outcomes [11–14]. For example, common SNPs in the T regulatory cell pathway gene, *TGFBR3*, have been associated with CRC survival [15]. Finally, evidence of strong genetic susceptibility variants for autoimmune and other immune-related diseases strengthens the premise for germline regulation of adaptive immune responses. To build upon prior studies, we conducted the largest and most comprehensive study to date of common germline genetic variability in relation to features of T cell responses in CRCs from population-based studies.

## Results

### GWAS of T cell receptor clonality

The discovery GWAS identified 18 SNPs with *p* < 5 × 10^− 6^ residing in 17 genomic regions associated with immunoSEQ-derived clonality. Of these, six variants at 2q24.1, 4q21.21, 6q21, 9p13.3, 10q25.2, and 18q21.32 remained below this statistical significance threshold upon joint discovery-replication meta-analysis (Table 1; Figure S2, S3). Although the associations from the joint analyses were mainly driven by the discovery data, the association results from the smaller replication sets still demonstrated the same direction and similar magnitude. The strongest association signal in the discovery phase arose from a common variant located at 10q25.2 [rs4918567; odds ratio (OR): 0.76 (95% confidence interval (CI): 0.69–0.84); *p* = 3.73 × 10^− 8^]. This variant remained associated with clonality in the joint meta-analyses at the genome-wide significant threshold with no evidence of heterogeneity (Table 1). eQTL analysis from healthy colon mucosa using BarcUVa-Seq showed that homozygous T/T individuals for rs4918567 had statistically significantly higher expression in *ADRA2A* (*p* = 0.012; Figure S4).

**Table 1:** Six novel loci associated with clonality from discovery GWAS and meta-analyses with *p* < 5 × 10^− 6^^†^

RS ID: CHR: BP	Nearest Gene	Locus	EFF/REF Allele*	Discovery Data (MECC)			Replication Data (CLX)			Replication Data (CRCGEN)			Meta Analysis			
				EAF^**^	OR (95% CI)^***^	p	EAF	OR (95% CI)	p	EAF	OR (95% CI)	p	EAF	OR (95% CI)	p	P_Heterogeneity_
rs4918567:10:112459918	*RBM20*	10q25.2	T/C	0.10	0.76 (0.69–0.84)	3.73E-08	0.19	0.90 (0.64–1.27)	0.5609	0.13	0.88 (0.63–1.24)	0.4594	0.21	0.78 (0.71–0.85)	4.90E-08	0.5029
rs34245610:18:56234676	*ALPK2*	18q21.32	T/C	0.81	1.20 (1.11–1.29)	9.46E-07	0.84	1.23 (0.83–1.83)	0.3049	0.82	1.15 (0.86–1.53)	0.3403	0.81	1.19 (1.12–1.28)	3.62E-07	0.9552
rs4443313:4:79040704	*FRAS1*	4q21.21	A/G	0.77	0.83 (0.78–0.89)	5.26E-08	0.71	0.83 (0.60–1.15)	0.2639	0.70	1.10 (0.88–1.38)	0.3820	0.76	0.85 (0.80–0.90)	3.75E-07	0.0548
rs18450843:6:106628943	*ATG5*	6q21	A/G	0.01	2.14 (1.57–2.91)	1.50E-06	0.01	1.44 (0.20-10.26)	0.7196	1.00	9.47 (0.99–90.78)	0.0531	0.01	2.17 (1.61–2.94)	4.97E-07	0.4043
rs76250771:2:155371283	*AC009227.3*	2q24.1	T/C	0.15	0.83 (0.77–0.90)	4.75E-06	0.08	0.81 (0.49–1.33)	0.4007	0.07	0.72 (0.47–1.11)	0.1367	0.15	0.83 (0.76–0.89)	1.31E-06	0.8111
rs7874748:9:35978632	*YBX1P10*	9p13.3	T/C	0.29	1.16 (1.09–1.23)	2.51E-06	0.34	1.12 (0.82–1.52)	0.4788	0.31	1.05 (0.84–1.32)	0.6530	0.33	1.15 (1.09–1.22)	2.49E-06	0.7057

* Effect/Reference Allele

^**^ Effect allele frequency

^***^ Odds ratio and 95% Confidence Intervals

^†^ Violin plots for best-call genotypes of each SNP and clonality are included in Supplementary Figure S19

A common variant residing in the intronic region of *FRAS1* at 4q21.21 [rs4443313; OR: 0.83 (95% CI: 0.78–0.89); *p* = 5.26 × 10^− 8^], approaching genome-wide statistical significance, was identified in the discovery phase. However, the strength of the association was weakened in the joint meta-analysis due to a different direction of association in one of the replication sets [P for heterogeneity = 0.055, Table 1]. This variant was associated with the differential expression of *LINC01094* in eQTL analysis using normal colon mucosa samples (*p* = 0.0025, Figure S5) and *CCNG2* in tumor tissue samples (*p* = 0.024, Figure S6).

A rare variant (rs184508436; MAF ranging from 0.5 to 0.9%) located at 6q21 was associated with clonality. Carrying the rare variant of rs184508436 was associated with increased clonality in the joint meta-analyses [OR: 2.17 (95% CI: 1.61–2.94), *p* = 4.97 × 10^− 7^; Table 1]. We did not find any noteworthy eQTL results for this variant.

The other association signals at 2q24.1, 9p13.3 and 18q21.32 involved common variants. Of note, SNP rs7874748 at 9p13.3 was an eQTL for several genes including *SPAG8* (using GTEx/v7 transverse colon), *RECK* and *NPR2* (healthy mucosa from BarcUVa-Seq; Figure S7). rs7874748 was also an eQTL for several genes including *RUSC2*, *CCDC107, CA9, TPM2, TLN1, MSMP*, *NPR2, OR13J1*, and *HRCT1* in tumor tissues (using Colonomics.org, Figure S8). In colorectal tumor tissues, SNP rs34245610 at 18q21.32 was an eQTL for *ATP8B1* (*p* = 0.010, Figure S9).

### GWAS of T cell receptor abundance

In the discovery phase, we identified 19 SNPs across 17 genomic regions associated with abundance with *p* < 5 × 10^− 6^. Of those, 11 SNPs remained with *p* < 5 × 10^− 6^ in the joint meta-analysis (Table 2; Figure S10, S11). The strongest association signal identified in the discovery phase was for a common variant (rs1518405) at 2q31.3 with a p value of 8.67 × 10^− 7^ (OR: 1.59, 95% CI: 1.32–1.91). However, no significant results for chromatin interactions or eQTL analysis were found. Of note, rs56148061 at 11q12 was associated with differential expression of *SYT7* in eQTL analysis using colorectal tumor samples (*p* = 0.007, Figure S12).

### GWAS of pathology-based tumor infiltrating lymphocytes (TILs)

**Fig. 1:**
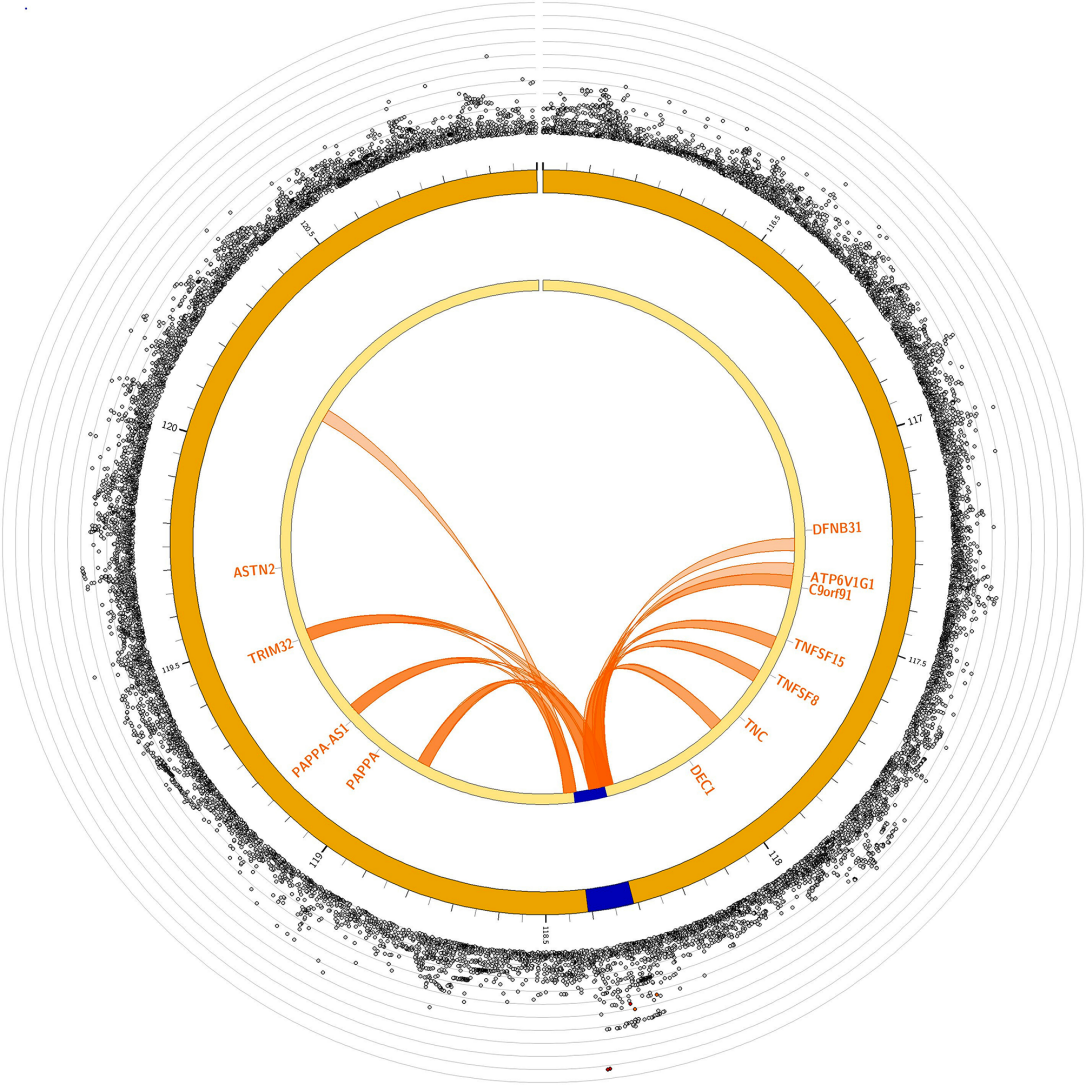
Chromatin interaction mapping results for TILs at 9q33. rs10982853 is associated with *DEC1*, *TNC*, *TNFSF8*, *PAPPA*, and *TRIM32*

In the discovery phase, we identified 6 SNPs with *p* < 5 × 10^− 6^ associated with the presence of TILs (Figure S13, S14). Two common variants at 9q33.1 and 20p13 remained with *p* < 5 × 10^− 6^ in joint meta-analyses (Table 3). rs10982853 at 9q33 was associated with TILs with a discovery p-value of 2.71 × 10^− 6^ (OR: 1.47, 95% CI: 1.25–1.72). Chromatin interaction mapping identified physical interactions with several local and distant genes including *TNC, TNFSF8*, *PAPPA-AS1*, and *TRIM32* (Fig. 1). eQTL analyses from healthy colon mucosa samples and GTEx/v8 showed statistically significant associations with *TNFSF8* (*p* = 0.0139, Figure S15). rs10982853 was associated with differential expression in *PAPPA* in eQTL analysis using tumor tissues (*p* = 0.028, Figure S16).

**Table 2:** Eleven novel loci associated with abundance from Discovery GWAS and Meta-analyses with *p* < 5 × 10^− 6^^†^

RSID: CHR: BP	Nearest Gene	Loci	EFF/REF Alleles^*^	Discovery (MECC)			Replication (CLX)			Replication (CRCGEN)			Meta Analysis			
				EAF^**^	OR (95% CI)^***^	p	EAF	OR (95% CI)^***^	p	EAF	OR (95% CI)^***^	p	EAF	OR (95% CI)^***^	p	P_Heterogeneity_
rs1518405:2:181016277	*CWC22*	2q31.3	A/T	0.97	1.59 (1.32–1.91)	8.67E-07	0.97	1.74 (0.74–4.07)	0.2054	0.99	0.97 (0.14–6.56)	0.9737	0.97	1.59 (1.33–1.90)	4.08E-07	0.8602
rs144595725:14:31526087	*AP4S1*	14q12	A/G	0.99	2.71 (1.82–4.03)	9.37E-07	NA	NA	NA	0.99	3.96 (0.18–88.64)	0.3871	0.99	2.73 (1.84–4.05)	6.11E-07	0.8129
rs56148061:11:61014040	*PGA4:PGA5*	11q12	A/C	0.04	0.60 (0.49–0.74)	1.30E-06	0.95	0.45 (0.18–1.08)	0.0766	0.95	2.45 (0.42–14.31)	0.3218	0.04	0.60 (0.50–0.74)	6.12E-07	0.2381
rs147844733:17:36994755	*C17orf98*	17q12	A/T	0.99	2.35 (1.68–3.28)	5.54E-07	NA	NA	NA	1.00	0.36 (0.01–19.56)	0.6191	0.99	2.32 (1.66–3.23)	6.99E-07	0.3604
rs142105062:11:122506047	*RP11-266E8.2*	11q13.1	T/C	0.01	0.17 (0.09–0.34)	4.29E-07	NA	NA	NA	0.01	1.04 (0.09–12.46)	0.9727	0.01	0.20 (0.10–0.38)	1.04E-06	0.1728
rs577783:6:137918440	*BTF3L4P3*	6q23.3	A/G	0.37	0.85 (0.80–0.91)	1.36E-06	0.45	1.08 (0.80–1.46)	0.6240	0.41	0.68 (0.45–1.03)	0.0722	0.63	0.86 (0.80–0.91)	1.23E-06	0.1791
rs9770081:7:622706	*PRKAR1B*	7p22.3	T/G	0.25	0.80 (0.74–0.88)	6.11E-07	NA	NA	NA	0.35	1.82 (0.92–3.62)	0.0887	0.25	0.81 (0.75–0.89)	2.01E-06	0.0206
rs12313375:12:94976499	*TMCC3*	12q22	A/C	0.98	0.58 (0.46–0.73)	4.56E-06	0.98	0.70 (0.23–2.10)	0.5270	0.99	0.29 (0.04–1.97)	0.2053	0.98	0.58 (0.46–0.73)	2.10E-06	0.7284
rs135429:22:44657217	*KIAA1644*	22q13.31	T/C	0.31	0.85 (0.79–0.91)	2.49E-06	0.36	0.86 (0.58–1.26)	0.4301	0.36	1.02 (0.66–1.58)	0.9114	0.31	0.85 (0.80–0.91)	2.43E-06	0.7032
rs4495475:8:66165989	*RPL31P41*	8q13.1	A/C	0.80	1.21 (1.12–1.30)	1.25E-06	0.82	1.03 (0.69–1.53)	0.8851	0.81	0.86 (0.49–1.51)	0.6014	0.80	1.19 (1.11–1.29)	2.73E-06	0.3817
rs190417185:2:74892755	*SEMA4F*	2p13.1	T/C	0.02	2.08 (1.53–2.82)	2.48E-06	NA	NA	NA	0.01	0 (0.00-3.94)	0.1030	0.02	2.06 (1.52–2.79)	3.18E-06	0.0703

* Effect/Reference Allele

^**^ Effect allele frequency

^***^ Odds ratio and 95% Confidence Intervals

^†^ Violin plots for best-call genotypes of each SNP and abundance are included in Supplementary Figure S19

**Table 3:** Two novel loci associated with TILs identified from Discovery GWAS and Meta-analyses with *p* < 5 × 10^− 6^

RS ID: CHR: BP	Nearest Gene	Loci	EFF/REF alleles^*^	Discovery (MECC)			Replication (Harvard Cohorts)			Replication (CLX)			Meta Analysis Results			
				EAF^**^	OR (95% CI)^***^	p	EAF	OR (95% CI)	p	EAF	OR (95% CI)	p	EAF	OR (95% CI)	p	P_Heterogeneity_
rs10982853:9:118405173	*RP11-284G10.1*	9q33.1	C/T	0.87	1.47 (1.25–1.72)	2.71E-06	0.87	1.22 (0.79–1.89)	0.3646	0.88	1.07 (0.40–2.83)	0.8928	0.87	1.43 (1.23–1.65)	2.85E-06	0.6272
rs215529:20:2767620	*RPL19P1*	20p13	C/T	0.57	0.77 (0.69–0.86)	1.53E-06	0.61	0.89 (0.66–1.19)	0.4238	0.54	1.33 (0.66–2.69)	0.4244	0.57	0.79 (0.72–0.87)	3.58E-06	0.2329

* Effect/Reference Allele

^**^ Effect allele frequency

^***^ Odds ratio and 95% Confidence Intervals

**Fig. 2:**
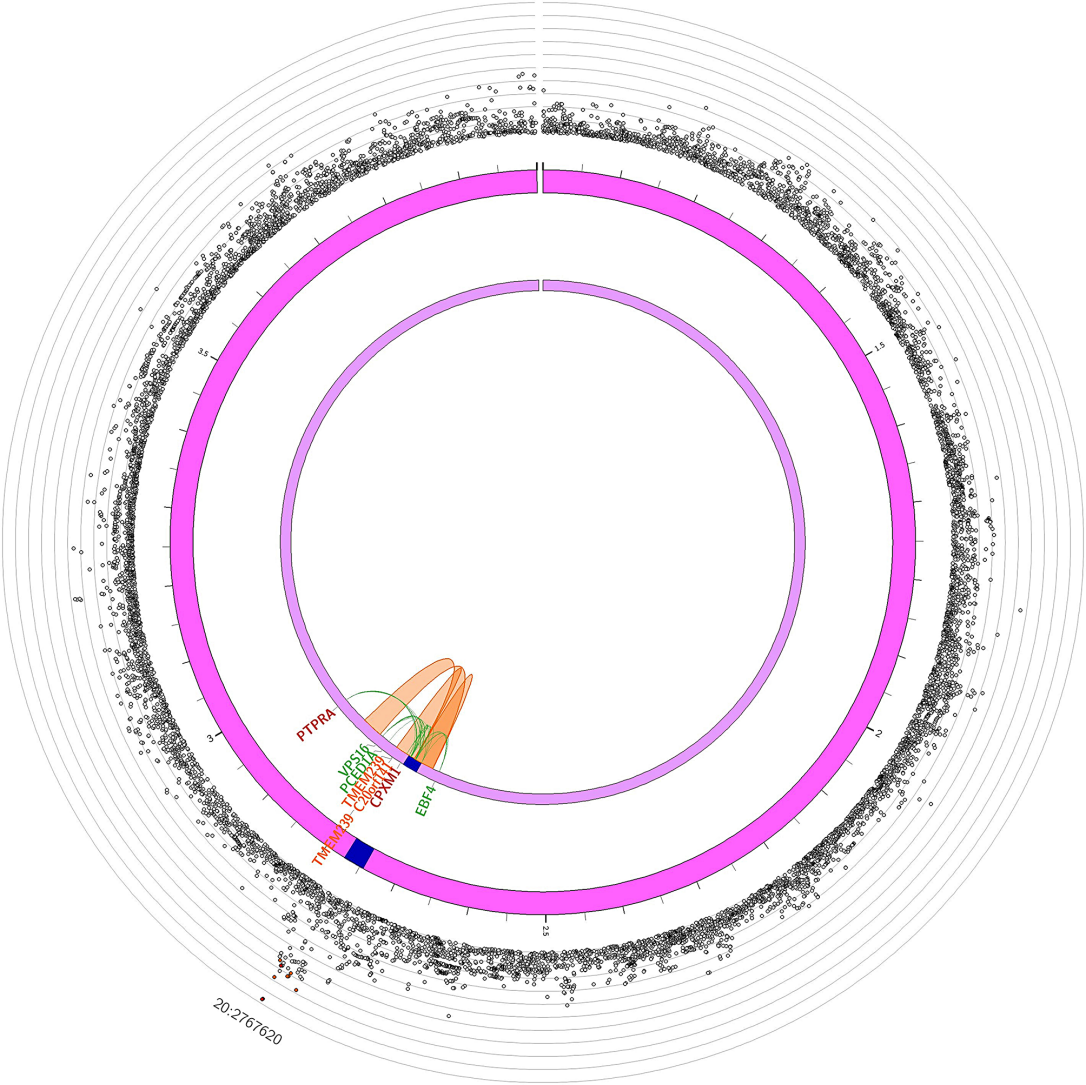
Chromatin interaction mapping results for TILs at 20p13. rs215529 is associated with *EBF4*, *VPS16*, *PCED1A*, *PTPRA*, and *CPXM1*

The SNP rs215529 at 20p13 was associated with TILs/hpf with a discovery p-value of 1.53 × 10^− 6^ (OR: 0.77, 95% CI: 0.69–0.86). Chromatin interactions were found with several genes including *EBF4* and *CPXM1* (Fig. 2). eQTL analyses using healthy colon mucosa samples showed significant associations with *FASTKD5* and *CPXM1* (*p* = 0.028, and 0.037, respectively, Figure S17). rs215529 was an eQTL for *PDYN* in tumor samples (*p* = 0.032, Figure S18).

### Subset analysis in MSS tumors

When restricting to MSS participants only, the directions of associations and significance levels remained consistent for the top 19 variants from the GWAS. Five additional SNPs were associated with *p* < 5 × 10^− 8^ specific to MSS tumors were identified (Table S2-S4).

### T cell subtype infiltration and top variants from GWAS

Of the 19 top variants from our GWAS analyses, one SNP was significantly associated with cytotoxic T cell infiltration as measured by mIF. rs1518405, with the strongest association for abundance (*p* = 4.08 × 10^− 7^), was significantly associated with the infiltration of CD3^+^/CD8^+^ T cells. Individuals who carry an A allele at this locus were associated with higher density of cytotoxic T cells (per allele OR: 1.54, 95% CI: 1.02–2.29, *p* = 0.0386), showing the same direction of association as we observed for abundance (OR: 1.59; 95% CI: 1.33–1.90). Another variant, rs577783 at 6q23.3, with a significant association in the joint meta-analysis for abundance (*p* = 1.23 × 10^− 6^), was associated with the infiltration of memory T cells (OR: 1.10, 95% CI: 1.01–1.20, *p* = 0.0316) and regulatory T cells (OR: 0.69, 95% CI: 0.49–0.97, *p* = 0.0351).

### Associations between previously reported variants with T cell features

Previously reported germline variants associated with immune infiltration in tumors were examined in the Discovery GWAS and the joint meta-analyses (Table S5). In a large study of the association between variants in immune related disorders and CRC risk, the authors reported two variants, rs11676348 and rs102275 [16]. Of those, we detected an association between rs102275, a susceptibility locus for Crohn’s disease, and abundance in the Discovery GWAS and joint meta-analyses (*p* = 0.0205 and 0.0061, respectively; Table S5). Another study demonstrated associations between two germline SNPs, rs3366 and rs4819959, and the amount of follicular helper T cells in all solid tumors using TCGA data [8]. These SNPs were not associated with any T cell features in our study. Lastly, of the 1,587 germline variants associated with 33 immune traits [9], we were able to examine 1,323 SNPs in our study but did not observe any associations between these variants and the T cell features in our data (Tables S6).

Common variants associated with CRC risk at a genome-wide significant threshold from previous GWAS were also investigated in relation to the three T cell features assessed in this study (Table S7). Among the 155 risk alleles, we identified 7, 9, and 7 SNPs (for clonality, abundance, and TILs, respectively) associated with T cell outcomes in the joint meta-analysis at a nominal level of statistical significance (*p* < 0.05).

## Discussion

Tumor immune microenvironments differ substantially between patients, yet the regulatory control of this diversity is not well understood. Here, we present evidence from a unique study of well-characterized, population-based, incident CRCs to explore inherited contributions to T cell responses to CRC. We showed that adaptive immune responses are partially determined by the inherited genome and identified specific candidate loci that contribute to both the intensity and diversity of T cell responses, as reflected by quantifiable measures of tumor infiltrating lymphocytes as well as specific clonal responses.

Our strongest association signal in the GWAS for T cell receptor clonality came from rs4918567 at 10q25.2 in the intronic region of *RBM20*. *RBM20*, encoding a protein that binds RNA and regulates splicing, is associated with familial cardiomyopathy [17]. A recent study indicates a potential role for *RBM20* in cardiovascular complications of diabetes by mediating insulin damage in cardiac tissues [18]. eQTL analyses for rs4918567 showed an association between homozygote T/T and higher expression in *ADRA2A*. *ADRA2A*, located downstream of *RBM20*, was associated with neutrophil percentage of leukocytes [19] and type 2 diabetes [20, 21]. Higher expression of *ADRA2A* was also associated with breast cancer survival and hypothesized to suppress cell proliferation and invasion through the PI3K/AKT/MTOR pathway in cervical cancer [22, 23]. Another variant rs4443313, located in the intronic region of *FRAS1*, was strongly associated with clonality. eQTL analyses for this SNP showed differential expression of *CCNG2* in colon tumor tissues.

We also identified a rare variant (rs184508436, MAF = 0.009), residing in the intergenic region of *ATG5* and *PRDM1*, to be strongly associated with clonality. *ATG5* encodes a protein involved in autophagic vesicle formation, negative regulation of innate antiviral immune response, and apoptosis [24]. A recent pan-cancer study using multiple public databases showed that the expression of *ATG5* is associated with tumor immune infiltration in most solid tumors [24]. *PRDM1* is a protein coding gene, which involves in the pathway of regulation of TP53 expression and degradation. SNPs in the region of *PRDM1* have been associated with Crohn’s disease and inflammatory bowel disease [25–28]. Although no significant eQTL results were identified for this variant, the potential role of *ATG5* in tumor metabolism and immune escape warrants further investigation.

The strongest association signal identified for T cell receptor abundance was rs1518405, located at 2q31.3 in the intergenic region of *CWC22*. Variants in *CWC22* have been associated with various traits, including schizophrenia, body mass index, and prostate cancer [29–31]. rs1518405 was also significantly associated specifically with the infiltration of CD3^+^/CD8^+^ T cells from our subset analysis using mIF. This preliminary finding provides evidence that the association between rs1518405 and abundance may be particularly driven by the infiltration of cytotoxic T cells.

We identified two variants associated with TILs. rs10982853, located in the intergenic region between *DELEC1* and *PAPPA* at 9q33, was significantly associated with several genes in this region from the eQTL and chromatic interaction mapping analyses. This region has been associated with blood protein levels and lymphocyte counts [19, 32–35]. *PAPPA* plays an important role in the activation of the IGF pathway. Overexpression of this protein may enhance IGF receptor signaling and promote tumor growth and invasion [36]. The second variant associated with TILs, rs215529, was located between a pseudogene *RPL19P1* and *CPXM1* at 20p13. *FASTKD5* and *CPXM1*, linked by chromatin interaction mapping, have been previously associated with white blood cell counts and blood protein levels [33, 35]. Interestingly, a recent study demonstrated that an immune-related gene prognostic index constructed using *SFRP4*, *CPXM1*, and *COL5A* was associated with better survival and better responses to immune checkpoint inhibitor therapy for head and neck squamous cell carcinoma [37]. *CPXM1* was also included in a tumor microenvironment prognostic signature profile comprised of 11 immune checkpoint genes for advanced-stage serous ovarian cancer [38]. 

Given that the tumor microenvironments in CRC differ by the MSI status, we analyzed the top variants from our study limited to the subset of patients with MSS tumors. When restricting analyses to patients with MSS tumors only, the top variants from our study showed the similar magnitude of effect sizes and p values remained significant (Table S2-S4). Furthermore, we performed analyses by treating MSI status as a confounder. Additional adjustment for MSI status in the discovery dataset did not yield any notable changes in the top findings (data not shown).

Upon examining germline variants identified in previous reports, we detected an association between rs102275 and T cell receptor abundance in our joint meta-analyses (*p* = 0.0061, Table S5). This variant at 11q12.2 is a risk SNP for Crohn’s Disease and inversely associated CRC risk (OR = 0.92, *p* = 2 × 10^− 5^).^16^ Our study showed that rs102275 may be associated with adaptive immune responses, especially the intensity of T cell infiltration. rs102275 was also located in proximity to rs174537 from 155 variants reported by a prior CRC GWAS (Table S7). rs174537 is significantly associated with CRC risk in individuals of East Asian and European descent (*p* = 9.22 × 10^− 21^ and 7.39 × 10^− 5^, respectively) [39]. It is also an eQTL for *FADS1* and *FADS2*. Although we did not observe an association for rs174537, rs102275 is in high LD with rs174537 (R^2^ = 0.933; D’=0.996) in European populations. Of note, rs102275 has been associated with several traits related to lipid metabolism and blood metabolite measurement from the NHGRI-EBI GWAS Catalog [40]. 

Our study is the first and largest population-based study compiling clinical, pathological and epidemiologic data with high-quality immunoSEQ results to investigate the association between germline genetic variation and T cell infiltration in CRC. Our findings suggest that germline genetic variation is associated with the quantity and quality of adaptive immune responses in CRC patients. To our knowledge, this unique study utilizes all existing relevant datasets and provides insight into the role of germline genetic variation in relation to the T cell repertoire, despite the replication dataset sample sizes. Nonetheless, we acknowlege that our study has several limitations. First, because the sample sizes of the three replication datasets were relatively small, the meta-analysis results were primarily driven by the discovery study. Likely due to the small sample sizes of the replication cohorts, most of the suggestive loci (*p* < 5 × 10^− 6^) from the discovery data were not independently replicated. Second, we recognize that using the conventional GWAS significance threshold (*p* < 5 × 10^− 8^) is a less stringent criterion for a larger number of imputed SNPs, including those with low-to-intermediate frequency, tested in our analyses. Importantly, here we present the combined results from all currently available epidemiological studies with genomic data and T cell immune features in CRC with the goal to highlight suggestive loci for the association between germline variations and T cell features in the CRC tumor microenvironment. Further studies with larger samples are warranted to replicate our findings and to identify the underlying functional genomic mechanisms. Because immune function is a key determinant of immunotherapy outcomes, there are important clinical implications of understanding the factors that influence the robustness and diversity of T cell responses in the CRC tumor microenvironment [41–44]. In CRC, several immune checkpoint inhibitors are FDA-approved for the treatment of metastatic microsatellite instable CRC, a molecular subtype of CRC in which TILs are enriched [43]. Our study underscores the importance of future work to determine the role of germline genetic regulation in response to immune checkpoint inhibitors. Germline genetic variation is likely to contribute to the wide range of responses to immune checkpoint inhibitors, and the results presented here provide direction for exploring candidate genes that are likely to regulate the adaptive immune responses in CRC. This presents the possibility that pharmacogenetic variation in the genes that regulate immune responses may have utility as predictive indicators for therapy.

## Methods

### Discovery Phase

#### Study Population

The Molecular Epidemiology of Colorectal Cancer Study (MECC) is a population-based study of incident CRC cases and healthy controls recruited from northern Israel from 1998 to 2017 [45]. Cases included patients with pathologically confirmed invasive colorectal adenocarcinomas. Controls are participants without a prior history of CRC selected from the same source population as cases and with individual matching on age, sex, Jewish ethnicity, and primary clinic site. Baseline demographic and clinical characteristics of the CRC cases contributing to this study are described in Table 4. Of note, the distributions of key demographic and clinical characteristics of all MECC cases and the subsets of cases whose tumors underwent pathological review and/or immunosequencing did not substantially differ (Table S1).

**Table 4:** Descriptive statistics for discovery and replication datasets

T Cell Repertoire Characteristics	Discovery		Replication					
	MECC		CLX		CRCGEN		NHS & HPFS	
**Abundance**	2395		92		162		-	
**Clonality**	2395		94		162		-	
**TILs**	2876		97		-		505	
**Cutoff**	0 vs. Any		< 1 vs. >=1				0 vs. Any	
	Mean	SD	Mean	SD	Mean	SD	Mean	SD
**Age**	69.5	12.2	70.9	9.03	67.60	10.04	69.3	8.84
	Count	%	Count	%	Count	%	Count	%
**Sex**								
Female	1383	48.1	28	28.9	61	62.4	250	49.5
Male	1493	51.9	69	71.1	101	37.7	255	50.5
**Race**								
White	2876	100.0	97	100.0	162	100.0	257	50.9
Other/Unknown	--	--	--	--	--	--	6	1.2
Missing	--	--	--	--	--	--	242	47.9
**Stage**								
I	467	16.2	0	0.0	0	0.0	139	27.5
II	1319	45.8	97	100.0	162	100.0	164	32.5
III	540	18.8	0	0.0	0	0.0	122	24.2
IV	302	10.5	0	0.0	0	0.0	42	8.3
Missing	250	8.7	0	0.0	0	0.0	38	7.5
**MSI Status**								
Stable	2249	78.2	97	100.0	162	100.0	372	73.6
Instable	417	14.5	0	0.0	0	0.0	67	13.3
Missing	210	7.3	--	--	--	--	66	13.1

#### Genotyping, quality control, and imputation and PCA analysis for discovery phase (MECC)

Germline DNA was extracted from peripheral blood samples. DNA samples from 10,364 MECC participants (5,581 CRC cases and 4,783 controls) were genotyped using four platforms. All genotype data were cleaned by platform using common quality control metrics at the individual and SNP levels as described previously [46–48]. 485 cases and 498 controls were genotyped in two batches using Illumina HumanOmni2.5 chips, measuring approximately 2.3 million SNPs [47]. Batch 1 (384 cases and 143 controls) was genotyped at the Case Western Reserve University, and batch 2 (101 cases and 355 controls) was genotyped at the University of Michigan. 1,155 cases and 1,117 controls were genotyped as part of the National Cancer Institute-sponsored Colorectal Transdisciplinary (CORECT) Study using a custom Affymetrix Axiom genome-wide platform measuring 1.2 million SNPs [48]. 3,768 cases and 3,028 controls were genotyped as part of the OncoArray Consortium using a custom Illumina OncoArray chip measuring 495 K SNPs (genome-wide backbone and known cancer susceptibility loci) [46]. The final set of MECC participants including 173 cases and 140 controls were genotyped on the commercialized Infinium OncoArray-500 K Beadchip (Figure S1).

We imputed genotypes to the Haplotype Reference Consortium (HRC) panel [49] (39.2 million variants) using the University of Michigan Imputation Server [50], separately by genotyping platform. Imputed SNPs with allele count less than 20 and genotypes with quality scores (Rsq) less than 0.3 from any of the platforms were excluded from downstream analyses.

Principal component analysis (PCA) was performed using PLINK 1.9^51^ on directly genotyped SNPs shared across the four genotyping panels: Illumina Omni2.5, Affymetrix Axiom, Illumina Custom OncoArray, and Illumina Infinium OncoArray-500 K. After LD pruning (r^2^ > 0.2), removing SNPs with minor allele frequency (MAF) < 0.01, and SNPs with PC1 and PC2 loading > 4.0, 55,852 autosomal SNPs were retained for PCA. Due to possible residual population substructure, the first 10 principal components for global ancestry were included in association analyses.

#### Quantification of tumor infiltrating lymphocytes

Hematoxylin and eosin–stained (H&E) tumor sections were blindly reviewed by a single gastrointestinal pathologist (J.K.G.) for 3,865 MECC participants. Tumor infiltrating lymphocytes (TILs) were evaluated as described previously [5]. The mean count of TILs/high powered field (hpf) for each tumor was calculated by taking the average number of TILs observed across five representative fields. Cases were separated into two groups (TILs/hpf = 0 or TILs/hpf > 0) for analyses.

#### T cell receptor repertoire characterization

A subset of the 3,865 CRC cases that underwent pathology review had sufficient macrodissected tissue and extracted DNA available for immunosequencing. After QC, the analytic dataset included 2,750 cases. T cell receptor clonality and abundance data were derived from immunoSEQ assays run by Adaptive Biotechnologies (Seattle, WA). ImmunoSEQ uses a multiplex PCR system to amplify hypervariable complementarity determining region 3β (CDR3β) sequences of the *TRB* gene (T cell receptor beta locus; https://www.genenames.org/data/gene-symbol-report/#!/hgnc_id/HGNC:12155). Clonality was estimated by a modified Simpson diversity index which quantifies the clonality and diversity of the amino acid sequences of the T cell receptors. Abundance was estimated using the normalized number of *TRB* reads divided by the estimate of the total number of cells. We performed log transformation of clonality and abundance data due to the right-skewed distributions of the raw data. For clonality, we calculated a z-transformation of each sample average based on the distribution of those that underwent the same version of the assay. Two versions of the immunoSEQ assay were used, V2 (*n* = 1,165) and V4 (*n* = 1,585). Since the distributions of clonality were different between the two versions, all of the analyses of clonality were stratified by version and meta-analyzed.

#### Statistical analyses

Regression analysis using imputed genotype dosage was conducted using PLINK v1.9 [51] to investigate the associations between each SNP and each of the three T cell features (clonality, abundance, TILs). All models were adjusted for sex, age, genotyping center, and global ancestry. For clonality, analyses were performed separately by immunoSEQ assay version, and summary statistics were combined using fixed-effect inverse variance-weighted meta-analysis implemented in METAL [52]. SNPs with a suggestive p value of less than 5 × 10^− 6^ from the discovery phase were further assessed in the replication datasets and in a joint meta-analysis.

### Replication phase

#### Study populations and analyses

Three independent data sets were available for replication of our discovery phase findings: Colonomics (“CLX”, *N* = 99; clonality, abundance, TILs/hpf), the CRC Genetics Study (“CRCGEN”, *N* = 162; clonality, abundance), and the Nurses’ Health Study and the Health Professionals Follow-up Study (*N* = 505; TILs). The first replication dataset was a previously described set of 99 patients with microsatellite stable colon cancer diagnosed at stage II with immunoSEQ data. 53, 54 The second replication data set (Colorectal Cancer Genetics Study; CRCGEN) included FFPE and frozen samples from 162 stage II microsatellite stable colon cancer patients with immunoSEQ data [54]. 

The third replication dataset was derived from two prospective cohort studies in the US: the Nurses’ Health Study and the Health Professionals Follow-up Study [13, 55]. Tissue sections for all CRC cases were examined by a single pathologist (S.O.). TILs were dichotomized into absent and present [2, 56]. The second pathologist (J.N.G.) independently re-reviewed 398 randomly selected cases, and a good inter-observer correlation was observed, as previously described [2].

#### Imputation, QC, and statistical analyses for replication data sets

Genotypes from CLX and CRCGEN were imputed to the Haplotype Reference Consortium (HRC) panel [49] and underwent similar QC to the discovery phase. Immunosequencing data for clonality and abundance from both replication sets underwent the same QC measures and transformations as the MECC discovery set. Regression models using imputed genotypes were conducted to examine the associations with T cell features for CLX and CRCGEN studies, adjusted for sex, age, and global ancestry.

Genotype data from the Harvard cohorts were imputed to the 1000 Genomes Project panel and underwent QC steps as described previously [13]. Logistic regression analyses were performed to investigate the association between each SNP and the presence of dichotomized TILs, after adjusting for sex, age, principal components, and tumor location.

### Meta-analysis

A meta-analysis for the discovery and replication phases on the three immune-related outcomes using fixed-effect models with inverse variance weighting was implemented in METAL [52]. Heterogeneity was evaluated using Cochran’s Q test for heterogeneity and the measure I^2^.

### Post-GWAS annotation

Functional Mapping and Annotation of Genome-Wide Association Studies (FUMA GWAS, https://fuma.ctglab.nl/) was used for post-GWAS analyses for the SNPs with a p-value less than 5 × 10^− 6^ in the discovery phase [57]. eQTL mapping and chromatin interaction mapping were conducted using various data sources including Genotype-Tissue Expression (GTEx) and Hi-C data from FUMA GWAS. For SNPs with discovery p values < 5 × 10^− 6^ for any of the three outcomes, additional eQTL analyses were performed as previously described using expression data on healthy colon mucosa samples (*N* = 485) from BarcUVa-Seq (https://barcuvaseq.org/) [58]. We also examined the associations between the 19 SNPs with p values < 5 × 10^− 6^ in joint meta-analyses and expression data from colorectal tumor tissues for genes located within 1 MB of the variants of interest using Colonomics (https://www.colonomics.org).[59]

### Subset analyses within microsatellite stable (MSS) tumors

To examine whether the top variants identified from our study were associated with T cell features independent of MSI status, we performed the same analyses restricted to patients with MSS tumors.

### Previously reported variants

We examined the associations between four germline variants associated with immune infiltration in tumors [8, 9] as well as 155 variants from previous GWAS of CRC risk [11–14] and immune-related outcomes in our Discovery GWAS and joint meta-analyses. We also examined 1,587 variants associated with 33 immune traits with suggestive *p* < 10^− 6^ from Sayaman et al. [9] in our discovery GWAS and joint meta-analyses.

### Multiplex immunofluorescence analysis

To further investigate the associations between the most statistically significant variants identified from our GWAS and the density of specific tumor-associated T cell populations, multiplex immunofluorescence (mIF) was performed on tissue microarray (TMA) blocks containing tumor cores from 357 MECC participants [60]. TMA sections were immunostained using PerkinElmer OPAL™ 7-Color Automation Immunohistochemistry (IHC) kits (Waltham, MA) on the BOND RX autostainer (Leica Biosystems, Vista, CA). Panel markers included CD3, CD4, CD8, FOXP3, CD45RO (PTPRC), KRT (keratin), and DAPI to stain nuclei. Immunostained slides were imaged with the Vectra3® Automated Quantitative Pathology Imaging System. Multispectral TIFF images were exported for quantitative image analyses in HALO (Indica Labs, New Mexico), and cell densities for each fluorescent marker in the nucleus and in the stroma as well as proportion of cells positive for a given marker or marker combination were generated. Here, we specifically examined the densities (cells/mm^2^) of cytotoxic T cells (CD3^+^/CD8^+^), memory T cells (CD3^+^/CD45RO^+^), and regulatory T cell (CD3^+^/CD4^+^/FOXP3^+^) populations. We performed the inverse hyperbolic sine transformation on the T cell density data to account for skewed distributions. The density of regulatory T cells was dichotomized into two groups (0 or any) due to the heavy-zero distribution.

Imputed genotypes from the 19 top variants from our GWAS were used to examine the association between SNPs and the infiltration of cytotoxic, memory, and regulatory (0 vs. any) T cells as implemented in regression models adjusting for sex, age, genotyping center, and global ancestry.

### Electronic supplementary material

Below is the link to the electronic supplementary material.

Supplementary Material 1

Supplementary Material 2

## Data Availability

Germline genetic data are available for other researchers through dbGAP for MECC study (https://www.ncbi.nlm.nih.gov/projects/gap/cgi-bin/study.cgi?study_id=phs001045.v1.p1; https://www.ncbi.nlm.nih.gov/projects/gap/cgi-bin/study.cgi?study_id=phs001856.v1.p1; https://www.ncbi.nlm.nih.gov/projects/gap/cgi-bin/study.cgi?study_id=phs001903.v1.p1) and HPFS/NHS studies (https://www.ncbi.nlm.nih.gov/projects/gap/cgi-bin/study.cgi?study_id=phs001078.v1.p1; https://www.ncbi.nlm.nih.gov/projects/gap/cgi-bin/study.cgi?study_id=phs001315.v1.p1), Colonomics (CLX) data can be found at https://www.colonomics.org/data-browser/. Data for eQTL analysis using BarcUVA can be found at https://barcuvaseq.org//cotrex/.

## Abbreviations

CRC: colorectal cancer
MECC: Molecular Epidemiology of Colorectal Cancer Study
TIL: tumor infiltrating lymphocyte
SNPs: Single nucleotide polymorphisms
GWAS: Genome-wide association study
MAF: Minor allele frequency
OR: Odds ratio
CI: Confidence interval
MSS: Microsatellite stable

## References

[1] Galon J, Costes A, Sanchez-Cabo F (2006). Type, density, and location of immune cells within human colorectal tumors predict clinical outcome. Science.

[2] Ogino S, Nosho K, Irahara N (2009). Lymphocytic reaction to colorectal cancer is associated with longer survival, independent of lymph node count, microsatellite instability, and CpG island methylator phenotype. Clin Cancer Res.

[3] Gooden MJ, de Bock GH, Leffers N (2011). The prognostic influence of tumour-infiltrating lymphocytes in cancer: a systematic review with meta-analysis. Br J Cancer.

[4] Huh JW, Lee JH, Kim HR (2012). Prognostic significance of tumor-infiltrating lymphocytes for patients with colorectal cancer. Arch Surg.

[5] Rozek LS, Schmit SL, Greenson JK, et al. Tumor-Infiltrating Lymphocytes, Crohn’s-Like Lymphoid Reaction, and Survival From Colorectal Cancer. J Natl Cancer Inst. 2016;108(8). 10.1093/jnci/djw027. 10.1093/jnci/djw027 PMC5017930 27172903

[6] Cozen W, Timofeeva MN, Li D (2014). A meta-analysis of Hodgkin lymphoma reveals 19p13.3 TCF3 as a novel susceptibility locus. Nat Commun.

[7] Yao S, Hong CC, Ruiz-Narvaez EA (2018). Genetic ancestry and population differences in levels of inflammatory cytokines in women: role for evolutionary selection and environmental factors. PLoS Genet.

[8] Shahamatdar S, He MX, Reyna MA et al. Germline Features Associated with Immune Infiltration in Solid Tumors. *Cell Rep* 2020;30(9):2900-08 e4. 10.1016/j.celrep.2020.02.039 [published Online First: 2020/03/05]. 10.1016/j.celrep.2020.02.039 PMC7082123 32130895

[9] Sayaman RW, Saad M, Thorsson V et al. Germline genetic contribution to the immune landscape of cancer. *Immunity* 2021;54(2):367– 86 e8. 10.1016/j.immuni.2021.01.011 [published Online First: 2021/02/11]. 10.1016/j.immuni.2021.01.011 PMC8414660 33567262

[10] Lim YW, Chen-Harris H, Mayba O (2018). Germline genetic polymorphisms influence tumor gene expression and immune cell infiltration. Proc Natl Acad Sci U S A.

[11] Law PJ, Timofeeva M, Fernandez-Rozadilla C (2019). Association analyses identify 31 new risk loci for colorectal cancer susceptibility. Nat Commun.

[12] Huyghe JR, Bien SA, Harrison TA (2019). Discovery of common and rare genetic risk variants for colorectal cancer. Nat Genet.

[13] Schmit SL, Edlund CK, Schumacher FR (2019). Novel common genetic susceptibility loci for Colorectal Cancer. J Natl Cancer Inst.

[14] Lu Y, Kweon SS, Tanikawa C (2019). Large-scale genome-wide Association study of East asians identifies loci Associated with Risk for Colorectal Cancer. Gastroenterology.

[15] Neumeyer S, Hua X, Seibold P (2020). Genetic Variants in the Regulatory T cell-related pathway and colorectal Cancer prognosis. Cancer Epidemiol Biomarkers Prev.

[16] Khalili H, Gong J, Brenner H (2015). Identification of a common variant with potential pleiotropic effect on risk of inflammatory bowel disease and colorectal cancer. Carcinogenesis.

[17] Guo W, Schafer S, Greaser ML (2012). RBM20, a gene for hereditary cardiomyopathy, regulates titin splicing. Nat Med.

[18] Liu J, Carnero-Montoro E, van Dongen J (2019). An integrative cross-omics analysis of DNA methylation sites of glucose and insulin homeostasis. Nat Commun.

[19] Vuckovic D, Bao EL, Akbari P et al. The Polygenic and Monogenic Basis of Blood Traits and Diseases. *Cell* 2020;182(5):1214-31 e11. 10.1016/j.cell.2020.08.008 [published Online First: 2020/09/06]. 10.1016/j.cell.2020.08.008 PMC7482360 32888494

[20] Totomoch-Serra A, Munoz ML, Burgueno J (2018). The ADRA2A rs553668 variant is associated with type 2 diabetes and five variants were associated at nominal significance levels in a population-based case-control study from Mexico City. Gene.

[21] Chen X, Liu L, He W (2013). Association of the ADRA2A polymorphisms with the risk of type 2 diabetes: a meta-analysis. Clin Biochem.

[22] Wang W, Guo X, Dan H (2020). alpha2A-Adrenergic receptor inhibits the progression of Cervical Cancer through blocking PI3K/AKT/mTOR pathway. Onco Targets Ther.

[23] Rivero EM, Martinez LM, Bruque CD (2019). Prognostic significance of alpha- and beta2-adrenoceptor gene expression in breast cancer patients. Br J Clin Pharmacol.

[24] Xu C, Zang Y, Zhao Y (2021). Comprehensive pan-cancer analysis confirmed that ATG5 promoted the Maintenance of Tumor Metabolism and the occurrence of Tumor Immune escape. Front Oncol.

[25] Anderson CA, Boucher G, Lees CW (2011). Meta-analysis identifies 29 additional ulcerative colitis risk loci, increasing the number of confirmed associations to 47. Nat Genet.

[26] Barrett JC, Hansoul S, Nicolae DL (2008). Genome-wide association defines more than 30 distinct susceptibility loci for Crohn’s disease. Nat Genet.

[27] de Lange KM, Moutsianas L, Lee JC (2017). Genome-wide association study implicates immune activation of multiple integrin genes in inflammatory bowel disease. Nat Genet.

[28] Liu JZ, van Sommeren S, Huang H (2015). Association analyses identify 38 susceptibility loci for inflammatory bowel disease and highlight shared genetic risk across populations. Nat Genet.

[29] Li Z, Chen J, Yu H (2017). Genome-wide association analysis identifies 30 new susceptibility loci for schizophrenia. Nat Genet.

[30] Tao S, Wang Z, Feng J (2012). A genome-wide search for loci interacting with known prostate cancer risk-associated genetic variants. Carcinogenesis.

[31] Zhu Z, Guo Y, Shi H (2020). Shared genetic and experimental links between obesity-related traits and asthma subtypes in UK Biobank. J Allergy Clin Immunol.

[32] Chen MH, Raffield LM, Mousas A et al. Trans-ethnic and Ancestry-Specific Blood-Cell Genetics in 746,667 Individuals from 5 Global Populations. *Cell* 2020;182(5):1198– 213 e14. 10.1016/j.cell.2020.06.045 [published Online First: 2020/09/06]. 10.1016/j.cell.2020.06.045 PMC7480402 32888493

[33] Emilsson V, Ilkov M, Lamb JR (2018). Co-regulatory networks of human serum proteins link genetics to disease. Science.

[34] Kanai M, Akiyama M, Takahashi A (2018). Genetic analysis of quantitative traits in the Japanese population links cell types to complex human diseases. Nat Genet.

[35] Sun BB, Maranville JC, Peters JE (2018). Genomic atlas of the human plasma proteome. Nature.

[36] Conover CA, Oxvig C (2018). PAPP-A and cancer. J Mol Endocrinol.

[37] Chen Y, Li ZY, Zhou GQ (2021). An Immune-Related Gene Prognostic Index for Head and Neck squamous cell carcinoma. Clin Cancer Res.

[38] Zheng M, Long J, Chelariu-Raicu A, et al. Identification of a Novel Tumor Microenvironment Prognostic signature for Advanced-Stage Serous Ovarian Cancer. Cancers (Basel). 2021;13(13). 10.3390/cancers13133343. [published Online First: 2021/07/21]. 10.3390/cancers13133343 PMC8268985 34283076

[39] Zhang B, Jia WH, Matsuda K (2014). Large-scale genetic study in East asians identifies six new loci associated with colorectal cancer risk. Nat Genet.

[40] Buniello A, MacArthur JAL, Cerezo M (2019). The NHGRI-EBI GWAS catalog of published genome-wide association studies, targeted arrays and summary statistics 2019. Nucleic Acids Res.

[41] Balar AV, Castellano D, O’Donnell PH (2017). First-line pembrolizumab in cisplatin-ineligible patients with locally advanced and unresectable or metastatic urothelial cancer (KEYNOTE-052): a multicentre, single-arm, phase 2 study. Lancet Oncol.

[42] Reck M, Rodriguez-Abreu D, Robinson AG (2016). Pembrolizumab versus Chemotherapy for PD-L1-Positive non-small-cell Lung Cancer. N Engl J Med.

[43] U.S. Food and Drug Administration: FDA grants accelerated approval to pembrolizumab for first tissue/site agnostic indication. [ www.fda.gov/Drugs/InformationOnDrugs/ApprovedDrugs/ucm560040.htm accessed October 24, 2018.

[44] Grosso J, Horak CE, Inzunza D (2013). Association of tumor PD-L1 expression and immune biomarkers with clinical activity in patients (pts) with advanced solid tumors treated with nivolumab (anti-PD-1; BMS-936558; ONO-4538). J Clin Oncol.

[45] Gruber SB, Moreno V, Rozek LS (2007). Genetic variation in 8q24 associated with risk of colorectal cancer. Cancer Biol Ther.

[46] Amos CI, Dennis J, Wang Z (2017). The OncoArray Consortium: A Network for understanding the Genetic Architecture of Common Cancers. Cancer Epidemiol Biomarkers Prev.

[47] Markowitz SD, Nock NL, Schmit SL (2016). A germline variant on chromosome 4q31.1 associates with susceptibility to developing Colon cancer metastasis. PLoS ONE.

[48] Schumacher FR, Schmit SL, Jiao S (2015). Genome-wide association study of colorectal cancer identifies six new susceptibility loci. Nat Commun.

[49] McCarthy S, Das S, Kretzschmar W (2016). A reference panel of 64,976 haplotypes for genotype imputation. Nat Genet.

[50] Das S, Forer L, Schonherr S (2016). Next-generation genotype imputation service and methods. Nat Genet.

[51] Purcell S, Neale B, Todd-Brown K (2007). PLINK: a tool set for whole-genome association and population-based linkage analyses. Am J Hum Genet.

[52] Willer CJ, Li Y, Abecasis GR (2010). METAL: fast and efficient meta-analysis of genomewide association scans. Bioinformatics.

[53] Sanz-Pamplona R, Berenguer A, Cordero D (2014). Aberrant gene expression in mucosa adjacent to tumor reveals a molecular crosstalk in colon cancer. Mol Cancer.

[54] Sanz-Pamplona R, Melas M, Maoz A (2020). Lymphocytic infiltration in stage II microsatellite stable colorectal tumors: a retrospective prognosis biomarker analysis. PLoS Med.

[55] Nishihara R, Wu K, Lochhead P (2013). Long-term colorectal-cancer incidence and mortality after lower endoscopy. N Engl J Med.

[56] Haruki K, Kosumi K, Li P (2020). An integrated analysis of lymphocytic reaction, tumour molecular characteristics and patient survival in colorectal cancer. Br J Cancer.

[57] Watanabe K, Taskesen E, van Bochoven A (2017). Functional mapping and annotation of genetic associations with FUMA. Nat Commun.

[58] Diez-Obrero V, Dampier CH, Moratalla-Navarro F (2021). Genetic effects on Transcriptome profiles in Colon epithelium provide functional insights for genetic risk loci. Cell Mol Gastroenterol Hepatol.

[59] Moreno V, Alonso MH, Closa A (2018). Colon-specific eQTL analysis to inform on functional SNPs. Br J Cancer.

[60] Kamal Y, Dwan D, Hoehn HJ (2021). Tumor immune infiltration estimated from gene expression profiles predicts colorectal cancer relapse. Oncoimmunology.

