# Supplementary material for: Germline genetic regulation of the colorectal tumor immune microenvironment
Source: BMC Genomics. 2024 Apr 25;25:409. doi: 10.1186/s12864-024-10295-1 (PMC11046907; doi:10.1186/s12864-024-10295-1)
Supplement: Supplementary file 2 — Supplementary Material 2 [file 12864_2024_10295_MOESM2_ESM.docx]

**Supplementary Tables and Figures**

*Table S1. Characteristics of all MECC cases and selected cases that underwent pathologic review and ImmunoSeq assays.*

| **Characteristic** | **All Cases** | | **GWAS and Pathology TILs/hpf Data** | | **GWAS and ImmunoSeq Data** | |
| --- | --- | --- | --- | --- | --- | --- |
|  | **Frequency** | **Percent** | **Frequency** | **Percent** | **Frequency** | **Percent** |
| **N** | 6,006 | 100.00 | 2,876 | 47.90 | 2,395 | 39.90 |
| **Age** | mean=68.8 | std=12.4 | mean=69.5 | std=12.2 | mean=69.4 | std=12.1 |
| **Sex** |  |  |  |  |  |  |
| Female | 2,869 | 47.77 | 1,385 | 48.12 | 1,153 | 48.10 |
| Male | 3,137 | 52.23 | 1,493 | 51.88 | 1,244 | 51.90 |
| **Ethnicity** |  |  |  |  |  |  |
| Arab | 898 | 14.95 | 365 | 12.68 | 287 | 11.97 |
| Ashkenazi | 3,572 | 59.47 | 1,805 | 62.72 | 1,507 | 62.87 |
| Sephardi | 1,190 | 19.81 | 549 | 19.08 | 465 | 19.40 |
| Other | 346 | 5.76 | 159 | 5.52 | 138 | 5.76 |
| **Jewish Ancestry** |  |  |  |  |  |  |
| Yes | 4,762 | 79.29 | 2,354 | 81.79 | 1,972 | 82.27 |
| No | 1,244 | 20.71 | 524 | 18.21 | 425 | 17.73 |
| **Stage** |  |  |  |  |  |  |
| I | 1,213 | 20.20 | 467 | 16.23 | 388 | 16.19 |
| II | 2,457 | 40.91 | 1,319 | 45.83 | 1,101 | 45.93 |
| III | 976 | 16.25 | 540 | 18.76 | 459 | 19.15 |
| IV | 631 | 10.51 | 302 | 10.49 | 252 | 10.51 |
| Missing | 729 | 12.14 | 250 | 8.69 | 197 | 8.22 |
| **MSI Status** |  |  |  |  |  |  |
| Stable | 3,256 | 82.16 | 2,249 | 84.36 | 1,939 | 86.02 |
| Instable | 707 | 17.84 | 417 | 18.54 | 315 | 13.98 |

*Table S2. Subset analysis on top SNPs for clonality in microsatellite stable (MSS) subjects. Three additional loci (denoted with* ***†****) were identified to be associated with clonality with p < 5x10^-8^ in patients with MSS tumors.*

| **RS ID: CHR:BP** | **Nearest Gene** | **Locus** | **EFF/REF Allele*** | **Discovery Data (MECC)**  **(N=1,938)** | | | **Replication Data (CLX)**  **(N=92)** | | | **Replication Data (CRCGEN)**  **(N=162)** | | | **Meta Analysis**  **(N=2,192)** | | | |
| --- | --- | --- | --- | --- | --- | --- | --- | --- | --- | --- | --- | --- | --- | --- | --- | --- |
|  |  |  |  | **EAF^**^** | **OR**  **(95% CI)^***^** | **p** | **EAF** | **OR (95% CI)** | **p** | **EAF** | **OR (95% CI)** | **p** | **EAF** | **OR (95% CI)** | **p** | **P_Heterogeneity_** |
| rs4918567:10:112459918 | *RBM20* | 10q25.2 | T/C | 0.10 | 0.77 (0.69-0.84) | 3.54E-06 | 0.19 | 0.90 (0.64-1.27) | 0.5609 | 0.13 | 0.88 (0.63-1.24) | 0.4594 | 0.22 | 0.79 (0.72-0.87) | 3.70E-06 | 0.5769 |
| rs34245610:18:56234676 | *ALPK2* | 18q21.32 | T/C | 0.81 | 1.19 (1.1-1.29) | 1.38E-05 | 0.84 | 1.23 (0.83-1.83) | 0.3049 | 0.82 | 1.15 (0.86-1.53) | 0.3403 | 0.81 | 1.19 (1.11-1.29) | 4.89E-06 | 0.9569 |
| rs4443313:4:79040704 | *FRAS1* | 4q21.21 | A/G | 0.77 | 0.82 (0.77-0.89) | 5.01E-07 | 0.71 | 0.83 (0.60-1.15) | 0.2639 | 0.70 | 1.10 (0.88-1.38) | 0.3820 | 0.76 | 0.85 (0.79-0.91) | 3.54E-06 | 0.0506 |
| rs184508436:6:106628943 | *ATG5* | 6q21 | A/G | 0.01 | 2.40 (1.72-3.34) | 2.32E-07 | 0.01 | 1.44 (0.20-10.26) | 0.7196 | 1.00 | 9.47 (0.99-90.78) | 0.0531 | 0.01 | 2.43 (1.76-3.36) | 6.55E-08 | 0.4336 |
| rs76250771:2:155371283 | *AC009227.3* | 2q24.1 | T/C | 0.15 | 0.81 (0.74-0.88) | 2.19E-06 | 0.08 | 0.81 (0.49-1.33) | 0.4007 | 0.07 | 0.72 (0.47-1.11) | 0.1367 | 0.15 | 0.80 (0.74-0.88) | 5.04E-07 | 0.8766 |
| rs7874748:9:35978632 | *YBX1P10* | 9p13.3 | T/C | 0.29 | 1.15 (1.08-1.24) | 3.85E-05 | 0.34 | 1.12 (0.82-1.52) | 0.4788 | 0.31 | 1.05 (0.84-1.32) | 0.6530 | 0.34 | 1.14 (1.07-1.22) | 3.51E-05 | 0.7357 |
| rs773042428:4:116966954**†** | *RP11-659O3.1* | 4q26 | C/G | 1.00 | 0.18 (0.1-0.33) | 4.35E-08 | - | - | - | - | - | - | 1.00 | 0.18 (0.1-0.33) | 3.83E-08 | 1 |
| rs149343558:6:106709461† | *ATG5* | 6q21 | T/C | 0.99 | 0.41 (0.3-0.58) | 1.76E-07 | 0.01 | 0.69 (0.1-5) | 0.718355 | 0.004 | 0.11 (0.01-0.94) | 0.0455478 | 0.99 | 0.41 (0.29-0.56) | 4.51E-08 | 0.4153 |
| rs75988446:1:191656812**†** | *RP11-309H21,2* | 1q31.2 | T/C | 1.00 | 0.03 (0.01-0.11) | 8.66E-07 | 0.01 | 0.08 (0.01-0.56) | 0.0122138 | - | - | - | 0.997 | 0.04 (0.01-0.13) | 4.24E-08 | 0.351 |

* Effect/Reference Allele

^**^ Effect allele frequency

^***^ Odds ratio and 95% Confidence Intervals

**†** Denote SNPs that are associated with clonality among patients with MSS tumors with p < 5x10^-8^.

*Table S3. Subset analysis on top SNPs for abundance in microsatellite stable (MSS) subjects. Two additional loci (denoted with* ***†****) were identified to be associated with abundance with p < 5x10^-8^ in patients with MSS tumors.*

| **RSID: CHR:BP** | **Nearest Gene** | **Loci** | **EFF/REF Alleles^*^** | **Discovery (MECC)**  **(N=1,938)** | | | **Replication (CLX)**  **(N=94)** | | | **Replication (CRCGEN)**  **(N=162)** | | | **Meta Analysis**  **(N=2,194)** | | | |
| --- | --- | --- | --- | --- | --- | --- | --- | --- | --- | --- | --- | --- | --- | --- | --- | --- |
|  |  |  |  | **EAF^**^** | **OR (95% CI)^***^** | **p** | **EAF** | **OR (95% CI)^***^** | **p** | **EAF** | **OR (95% CI)^***^** | **p** | **EAF** | **OR (95% CI)^***^** | **p** | **P_Heterogeneity_** |
| rs1518405:2:181016277 | CWC22 | 2q31.3 | A/T | 0.970 | 1.59 (1.31-1.94) | 4.22E-06 | 0.032 | 1.74 (0.74-4.07) | 0.2054 | 0.012 | 0.97 (0.14-6.56) | 0.9737 | 0.970 | 1.60 (1.32-1.94) | 1.73E-06 | 0.8417 |
| rs144595725:14:31526087 | AP4S1 | 14q12 | A/G | 0.991 | 2.55 (1.69-3.84) | 7.94E-06 | - | - | - | 0.007 | 3.96 (0.18-88.64) | 0.3871 | 0.991 | 2.55 (1.69-3.84) | 7.50E-06 | 1.0000 |
| rs56148061:11:61014040 | PGA4:PGA5 | 11q12 | A/C | 0.038 | 0.58 (0.46-0.72) | 1.39E-06 | 0.954 | 0.45 (0.18-1.08) | 0.0766 | 0.950 | 2.45 (0.42-14.31) | 0.3218 | 0.039 | 0.57 (0.46-0.71) | 2.88E-07 | 0.5704 |
| rs147844733:17:36994755 | C17orf98 | 17q12 | A/T | 0.991 | 2.00 (1.36-2.93) | 4.35E-04 | - | - | - | 0.003 | 0.36 (0.01-19.56) | 0.6191 | 0.991 | 2 (1.36-2.93) | 4.25E-04 | 1.0000 |
| rs142105062:11:122506047 | RP11-266E8.2 | 11q13.1 | T/C | 0.005 | 0.21 (0.1-0.44) | 2.66E-05 | - | - | - | 0.986 | 1.04 (0.09-12.46) | 0.9727 | 0.005 | 0.21 (0.1-0.44) | 2.54E-05 | 1.0000 |
| rs577783:6:137918440 | BTF3L4P3 | 6q23.3 | A/G | 0.370 | 0.82 (0.77-0.89) | 1.56E-07 | 0.549 | 1.08 (0.8-1.46) | 0.6240 | 0.594 | 0.68 (0.45-1.03) | 0.0722 | 0.380 | 0.84 (0.78-0.9) | 5.53E-07 | 0.0890 |
| rs9770081:7:622706 | PRKAR1B | 7p22.3 | T/G | 0.252 | 0.80 (0.73-0.88) | 2.85E-06 | - | - | - | 0.650 | 1.82 (0.92-3.62) | 0.0887 | 0.252 | 0.80 (0.73-0.88) | 2.66E-06 | 1.0000 |
| rs12313375:12:94976499 | TMCC3 | 12q22 | A/C | 0.981 | 0.50 (0.39-0.65) | 1.90E-07 | 0.020 | 0.70 (0.23-2.1) | 0.5270 | 0.012 | 0.29 (0.04-1.97) | 0.2053 | 0.981 | 0.51 (0.4-0.66) | 1.65E-07 | 0.5671 |
| rs135429:22:44657217 | KIAA1644 | 22q13.31 | T/C | 0.685 | 0.86 (0.8-0.92) | 4.44E-05 | 0.361 | 0.86 (0.58-1.26) | 0.4301 | 0.356 | 1.02 (0.66-1.58) | 0.9114 | 0.673 | 0.86 (0.8-0.92) | 3.06E-05 | 0.9996 |
| rs4495475:8:66165989 | RPL31P41 | 8q13.1 | A/C | 0.208 | 1.20 (1.1-1.3) | 2.05E-05 | 0.821 | 1.03 (0.69-1.53) | 0.8851 | 0.810 | 0.86 (0.49-1.51) | 0.6014 | 0.234 | 1.19 (1.1-1.29) | 2.57E-05 | 0.4609 |
| rs190417185:2:74892755 | SEMA4F | 2p13.1 | T/C | 0.013 | 2.19 (1.52-3.14) | 2.55E-05 | - | - | - | 0.994 | 0 (0-3.94) | 0.1030 | 0.013 | 2.19 (1.52-3.14) | 2.44E-05 | 1.0000 |
| rs533820043:4:140253161**†** | NAA15 | 4q31 | T/C | 0.006 | 0.22 (0.13-0.37) | 2.08E-08 | - | - | - | - | - | - | 0.006 | 0.22 (0.13-0.37) | 1.82E-08 | 1.0000 |
| rs571849233:4:140353143**†** | RP11-83A24.2 | 4q31 | T/C | 0.006 | 0.23 (0.14-0.39) | 4.47E-08 | - | - | - | - | - | - | 0.006 | 0.23 (0.14-0.39) | 3.95E-08 | 1 |

* Effect/Reference Allele

^**^ Effect allele frequency

^***^ Odds ratio and 95% Confidence Intervals.

**†** Denote SNPs that are associated with abundance among patients with MSS tumors with p < 5x10^-8^.

*Table S4. Subset analysis on top SNPs for TILs in microsatellite stable (MSS) subjects.*

| **RS ID: CHR:BP** | **Nearest Gene** | **Loci** | **EFF/REF alleles^*^** | **Discovery (MECC)**  **(N=2,248)** | | | **Replication (CLX)**  **(N=97)** | | | **Meta Analysis Results**  **(N=2,345)** | | | |
| --- | --- | --- | --- | --- | --- | --- | --- | --- | --- | --- | --- | --- | --- |
|  |  |  |  | **EAF^**^** | **OR (95% CI)^***^** | **p** | **EAF** | **OR (95% CI)** | **p** | **EAF** | **OR (95% CI)** | **p** | **P_Heterogeneity_** |
| rs10982853:9:118405173 | RP11-284G10.1 | 9q33.1 | C/T | 0.133 | 1.51 (1.26-1.81) | 8.40E-06 | 0.88 | 1.07 (0.40-2.83) | 0.8928 | 0.132 | 1.49 (1.25-1.78) | 1.06E-05 | 0.4969 |
| rs215529:20:2767620 | RPL19P1 | 20p13 | C/T | 0.431 | 0.77 (0.69-0.87) | 2.66E-05 | 0.54 | 1.33 (0.66-2.69) | 0.4244 | 0.432 | 0.78 (0.7-0.88) | 6.17E-05 | 0.1348 |

* Effect/Reference Allele

^**^ Effect allele frequency

^***^ Odds ratio and 95% Confidence Intervals.

*Table S5. SNPs associated with tumor immune infiltration from previous literature.*

| **rsID** | **Chr:Pos (EFF/REF allele)** | **MAF/Odds Ratio/P value** | **Reported Previously** | **Genotyped or Imputed in Discovery GWAS** | **TCR Clonality** | | **TCR Abundance** | | **TILs/hpf** | |
| --- | --- | --- | --- | --- | --- | --- | --- | --- | --- | --- |
|  |  |  |  |  | **Discovery** | **Meta-Analysis** | **Discovery** | **Meta-Analysis** | **Discovery** | **Meta-Analysis** |
| rs11676348 | 2:219010146 | MAF | 0.5 | Genotyped | 0.57 | 0.56 | 0.57 |  | 0.56 |  |
| (Carcinogenesis, 2015) | T/C | OR |  |  | 0.95 | 0.98 | 0.98 | 1.04 | 1.02 | 1.00 |
|  |  | **P** | **7.00E-05** |  | **0.0692** | **0.3626** | **0.6195** | **0.2600** | **0.7002** | **0.9460** |
|  |  |  |  |  |  |  |  |  |  |  |
| rs102275 | 11:61557803 | MAF |  | Genotyped | 0.29 | 0.29 | 0.29 |  | 0.29 |  |
| (Carcinogenesis, 2015) | T/C | OR |  |  | 0.95 | 0.96 | 0.92 | 0.91 | 0.94 | 0.95 |
|  |  | **P** | **2.00E-05** |  | **0.1266** | **0.1432** | **0.0205** | **0.0061** | **0.2547** | **0.3221** |
| rs3366 | 21:44834825 | MAF |  | Genotyped in Omni only | 0.24 | 0.28 | 0.24 |  | 0.24 |  |
| (Cell Report, 2020) | G/A | OR |  |  | 0.94 | 0.95 | 1.02 | 0.99 | 1.01 | 0.99 |
|  |  | **P** | **2.99E-09** |  | **0.0917** | **0.1686** | **0.6095** | **0.7430** | **0.9166** | **0.8944** |
|  |  |  |  |  |  |  |  |  |  |  |
| rs4819959 | 22:17586631 | MAF |  | Imputed | 0.50 | 0.50 | 0.50 |  | 0.50 |  |
| (Cell Report, 2020) | G/A | OR |  |  | 1.02 | 1.03 | 1.04 | 0.98 | 0.99 | 1.01 |
|  |  | **P** | **2.52E-16** |  | **0.6149** | **0.3216** | **0.2545** | **0.4369** | **0.7896** | **0.8465** |

Figure S1. Detailed information on genotyping and SNP quality control for the Discovery GWAS.


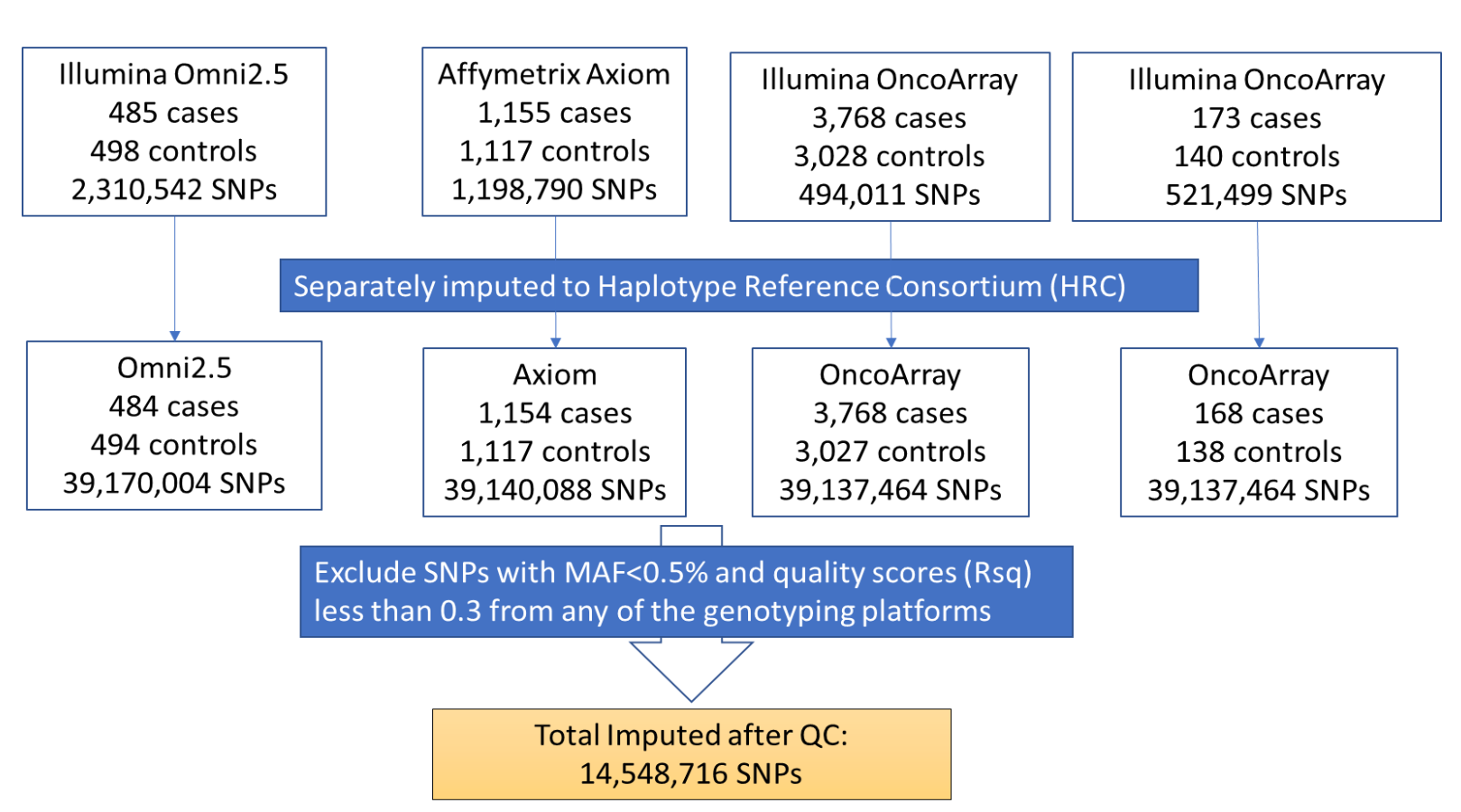


Figure S2. Q-Q plot for clonality from Discovery GWAS (N_snp_=8,775,871).


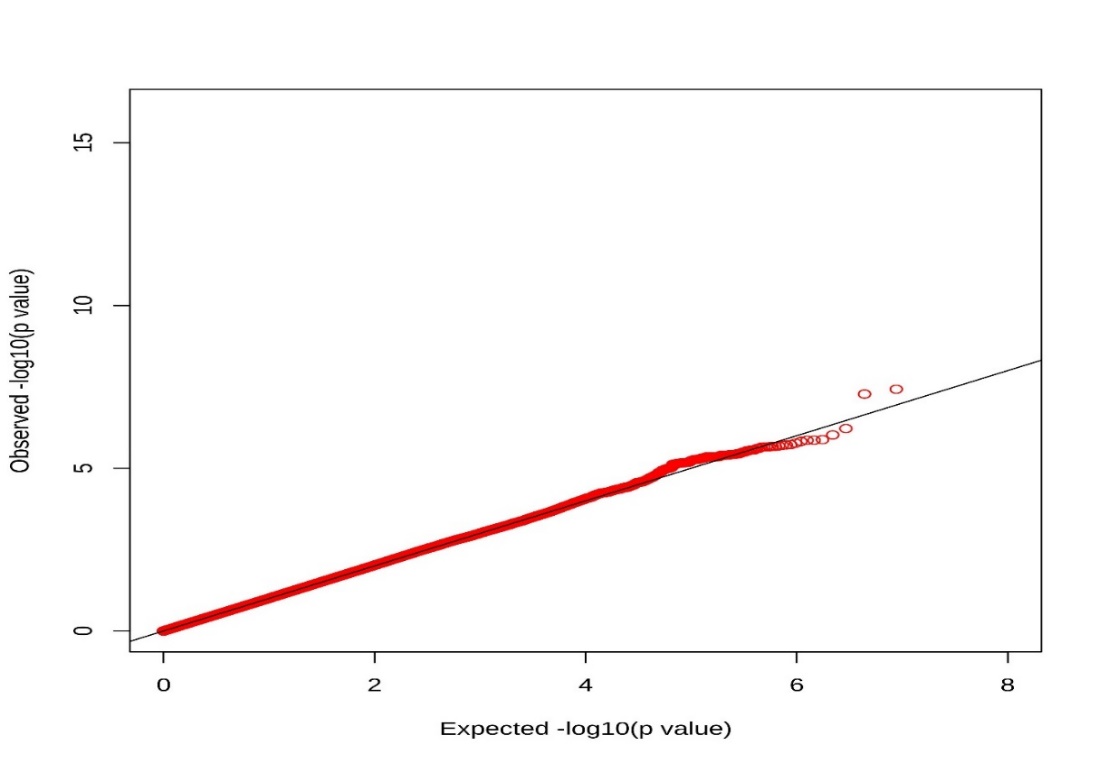


Figure S3. Manhattan plot for clonality from Discovery GWAS (N_snp_=8,775,871).


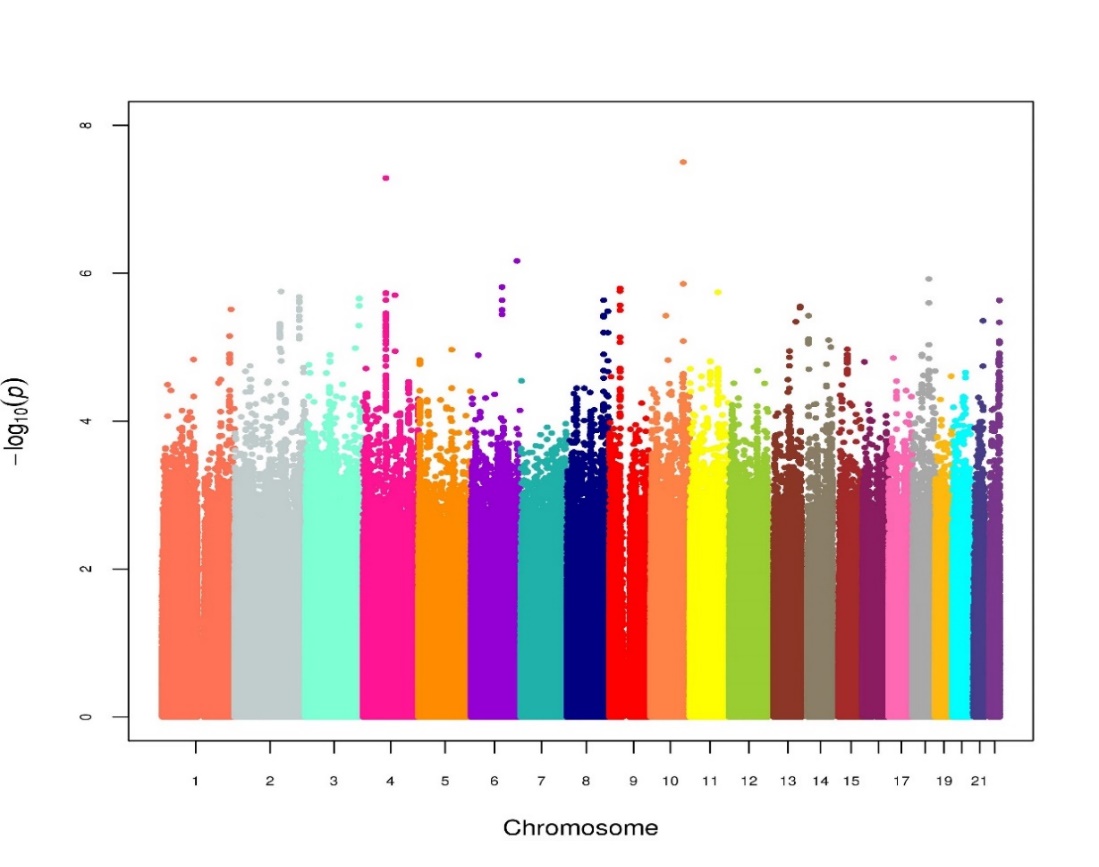


Figure S4. eQTL results for rs4918567 with ADRA2A using BarcUV-Seq healthy colonic mucosa transcriptomic data.

P=0.0119

Figure S5. eQTL results for rs4443313 with LINC01094 using BarcUVa-Seq healthy colonic mucosa transcriptomic data.

P=0.0025

*Figure S6. eQTL result for rs4443313 with CCNG2 using Colonomics colon tumor tissue transcriptomic data.*


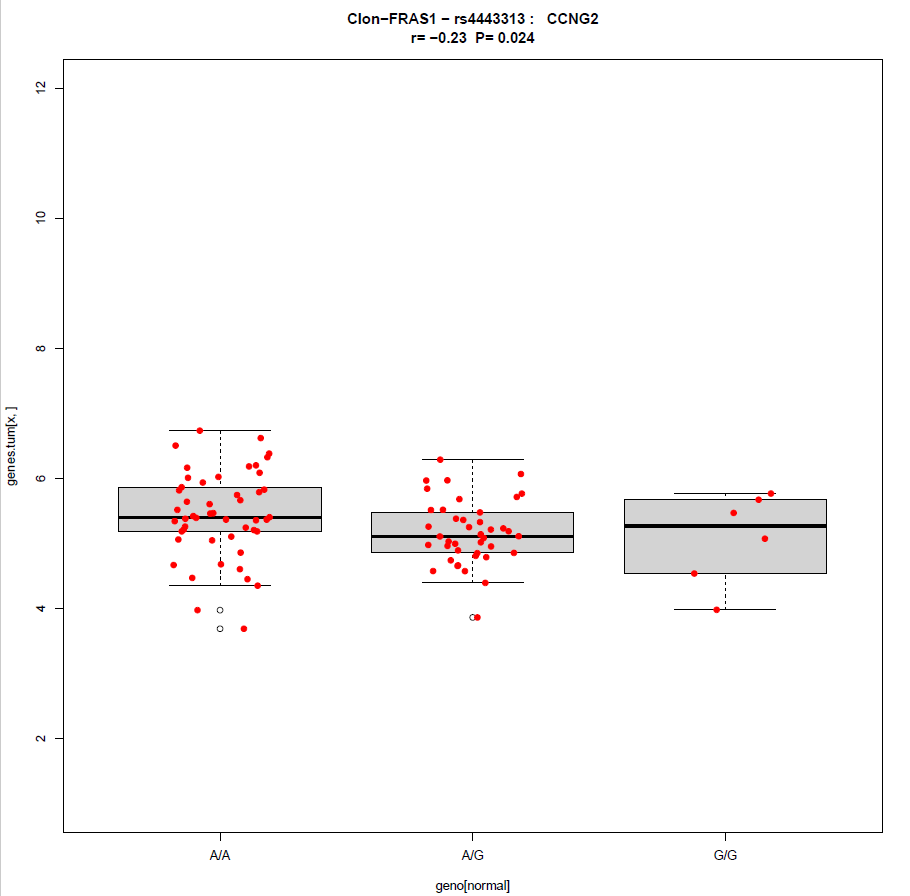


Figure S7. eQTL results for rs7874748 with NPR2 and RECK using BarcUVa-Seq healthy colonic mucosa transcriptomic data.

P=0.0497

P=0.0269

*Figure S8. eQTL result for rs7874748 with ALPK2 and NEDD4L using Colonomics colon tumor tissue transcriptomic data.
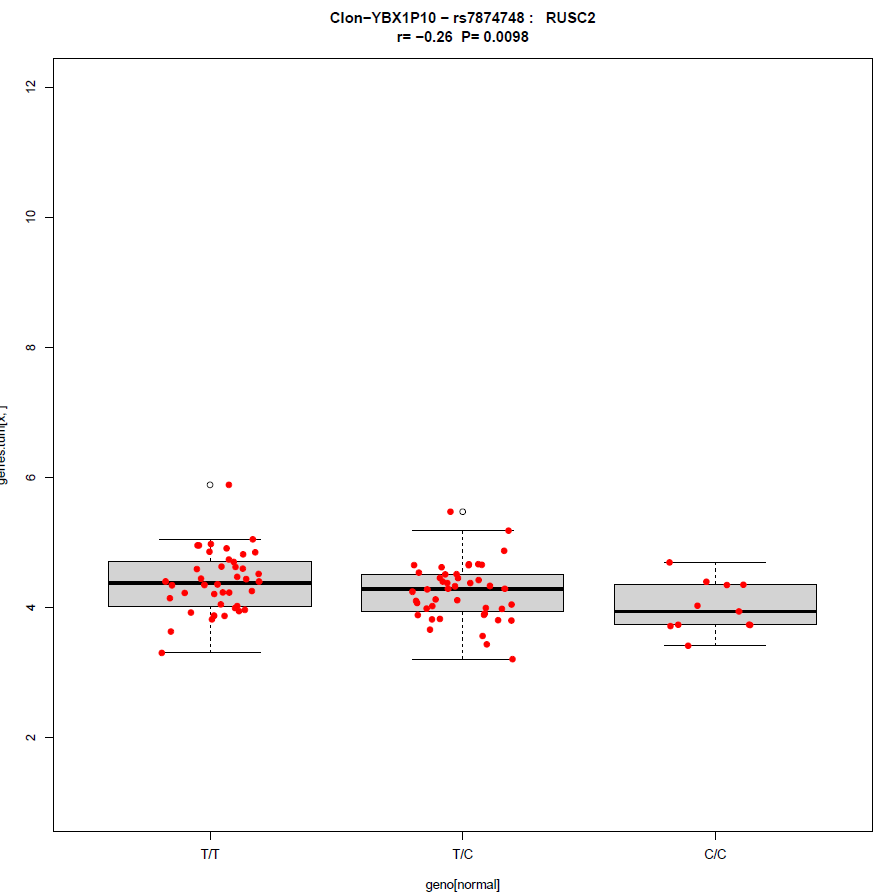

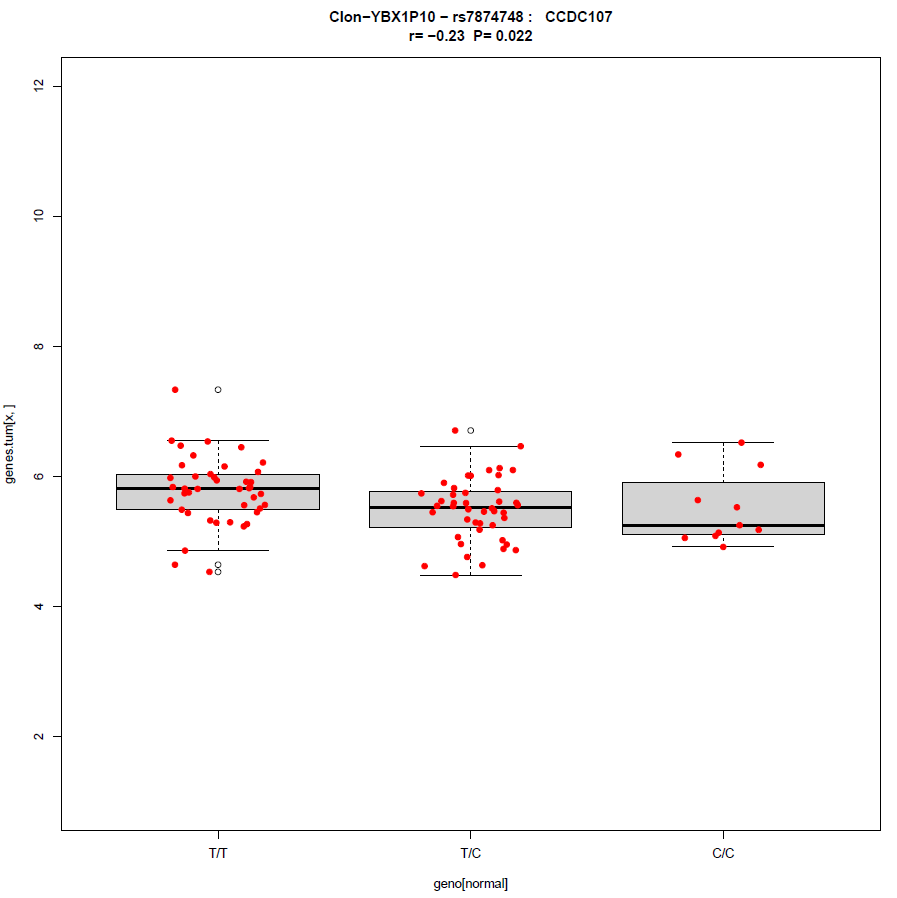
* *
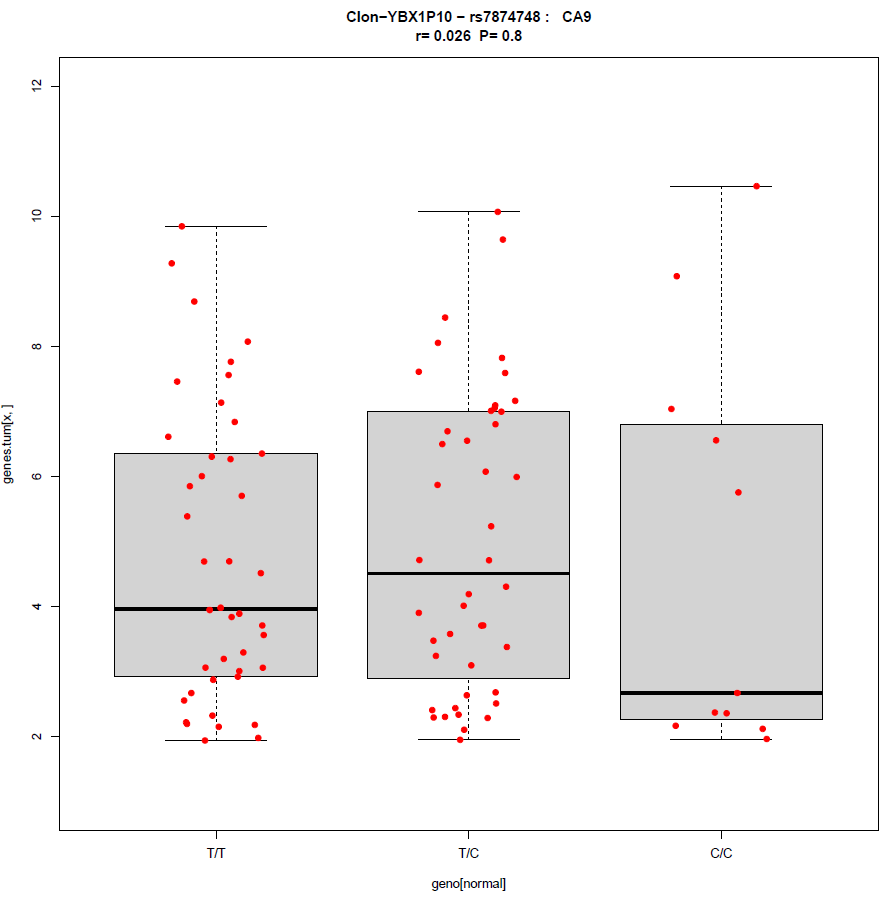
*
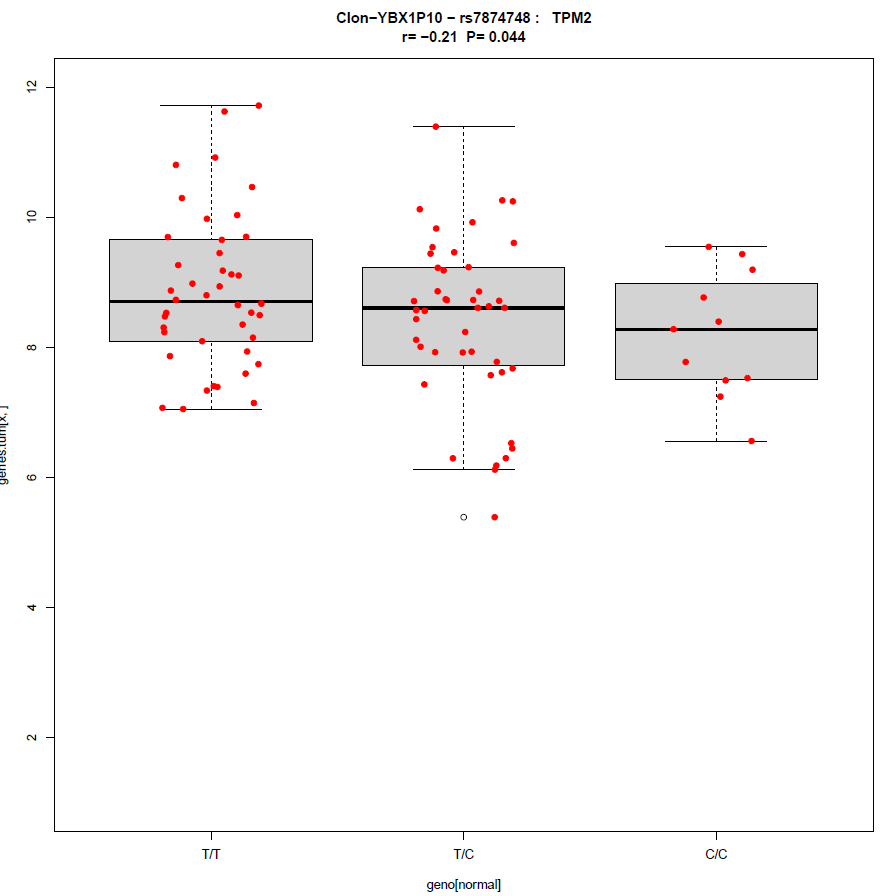


*
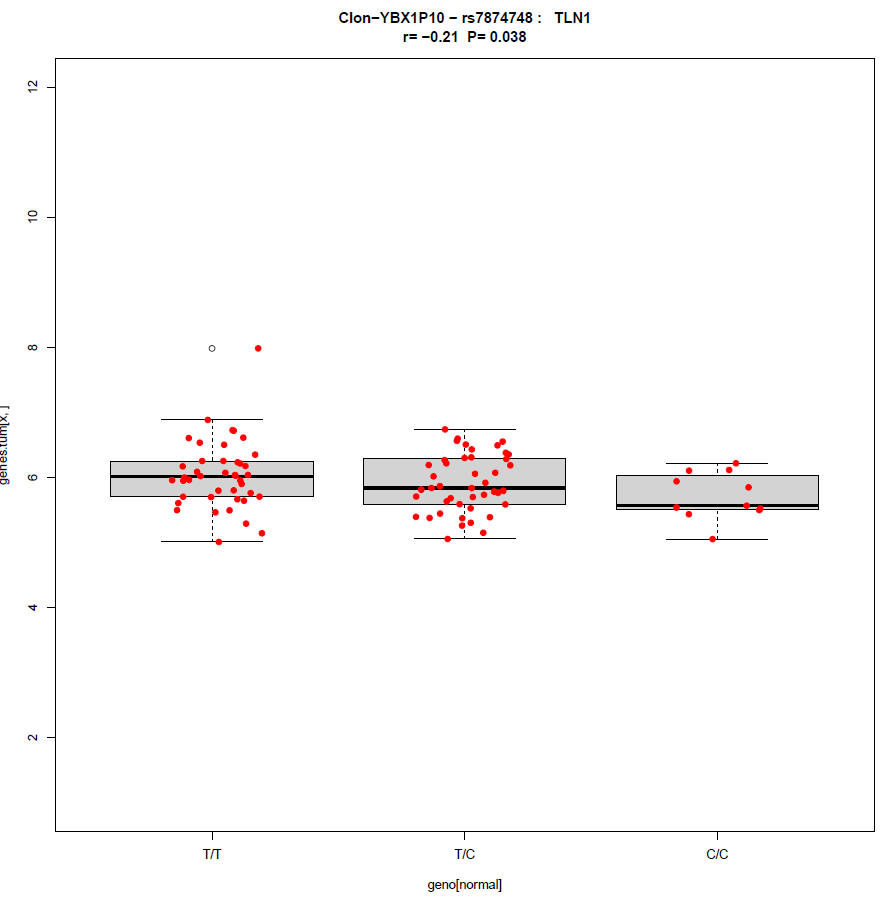

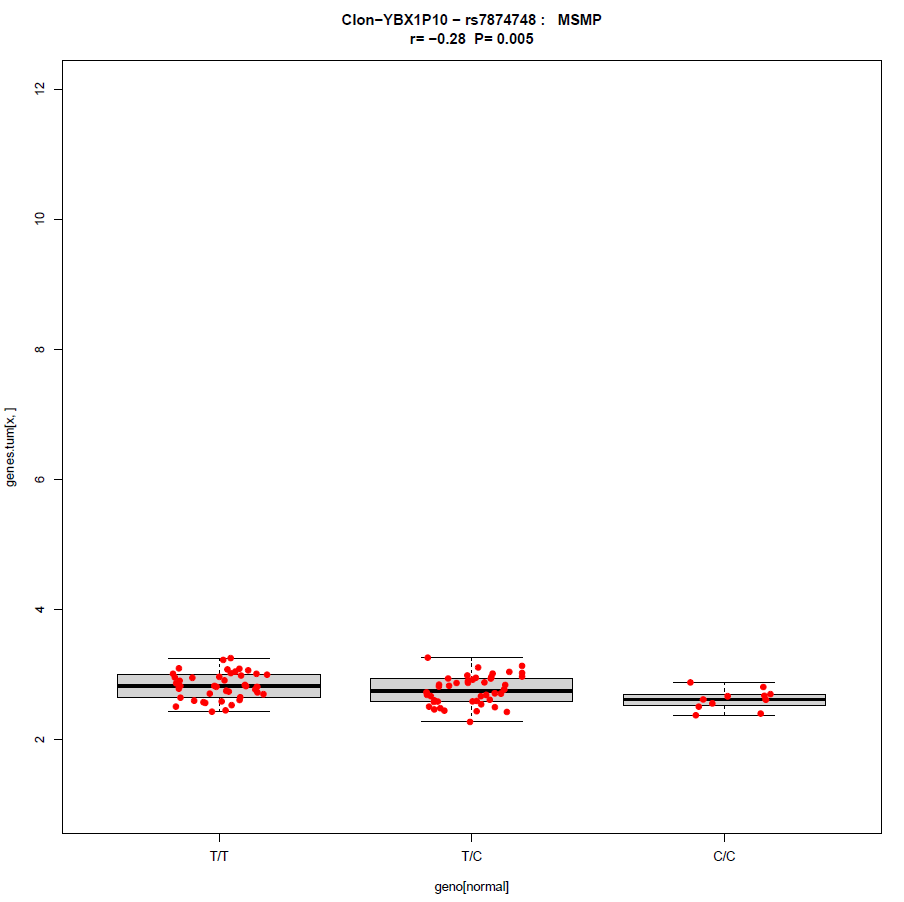

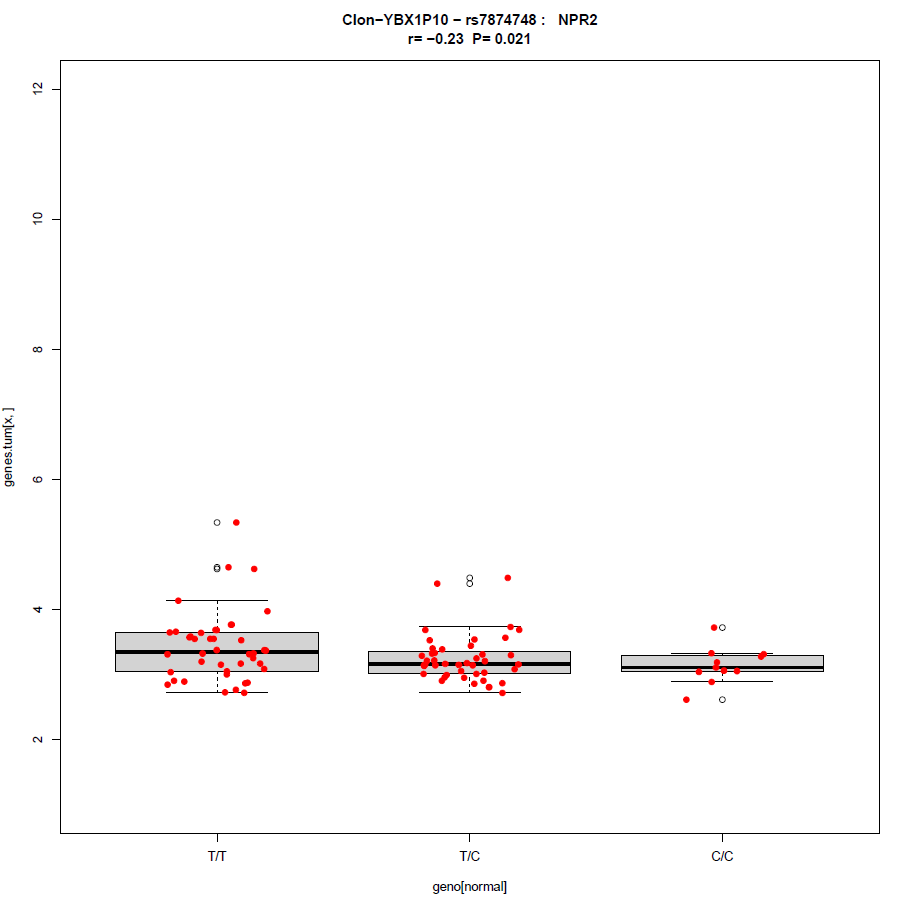

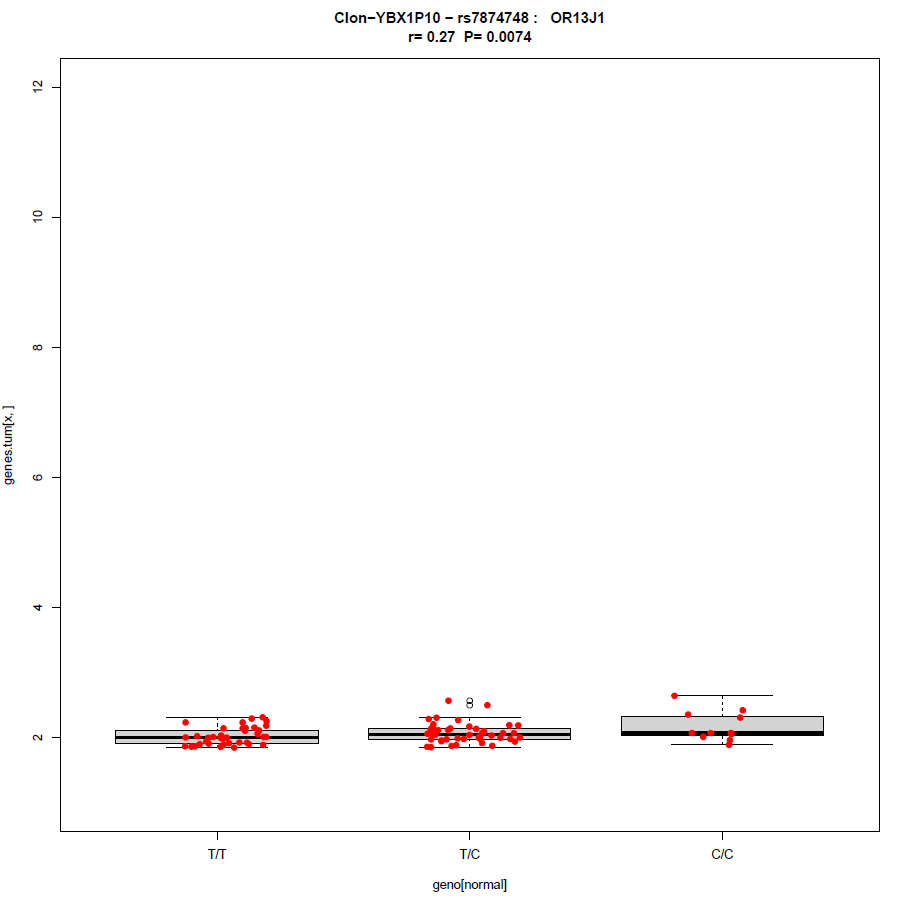

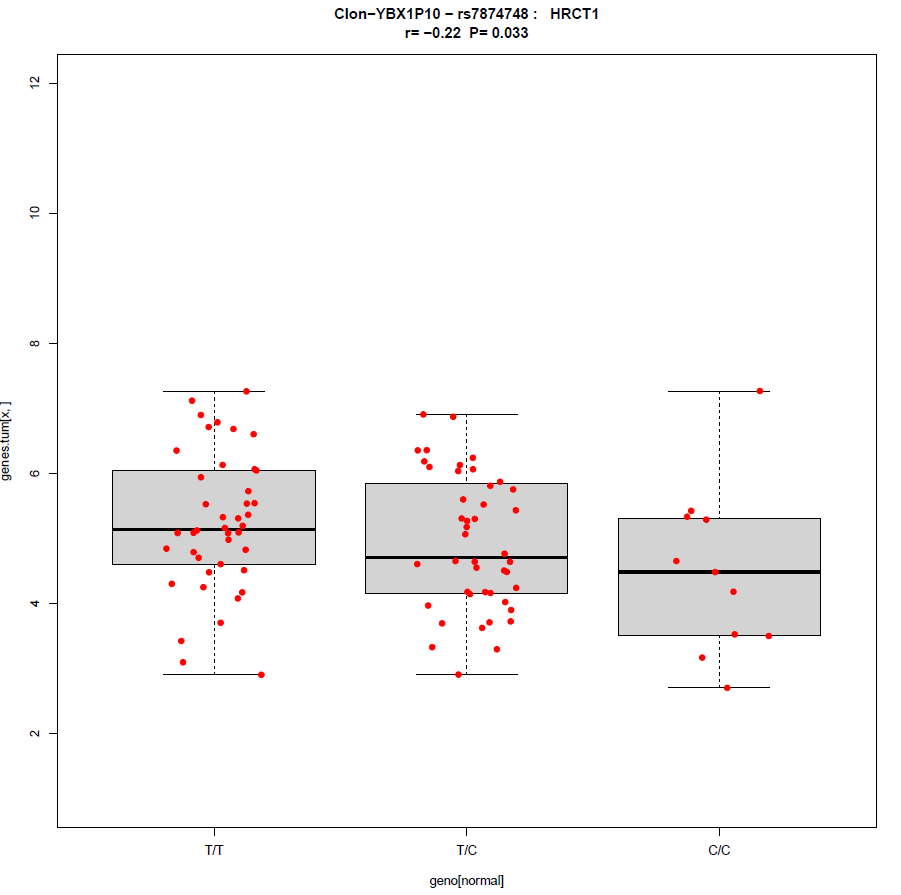
*

Figure S9. eQTL result for rs34245610 with ATP8B1 using Colonomics colon tumor tissue transcriptomic data.


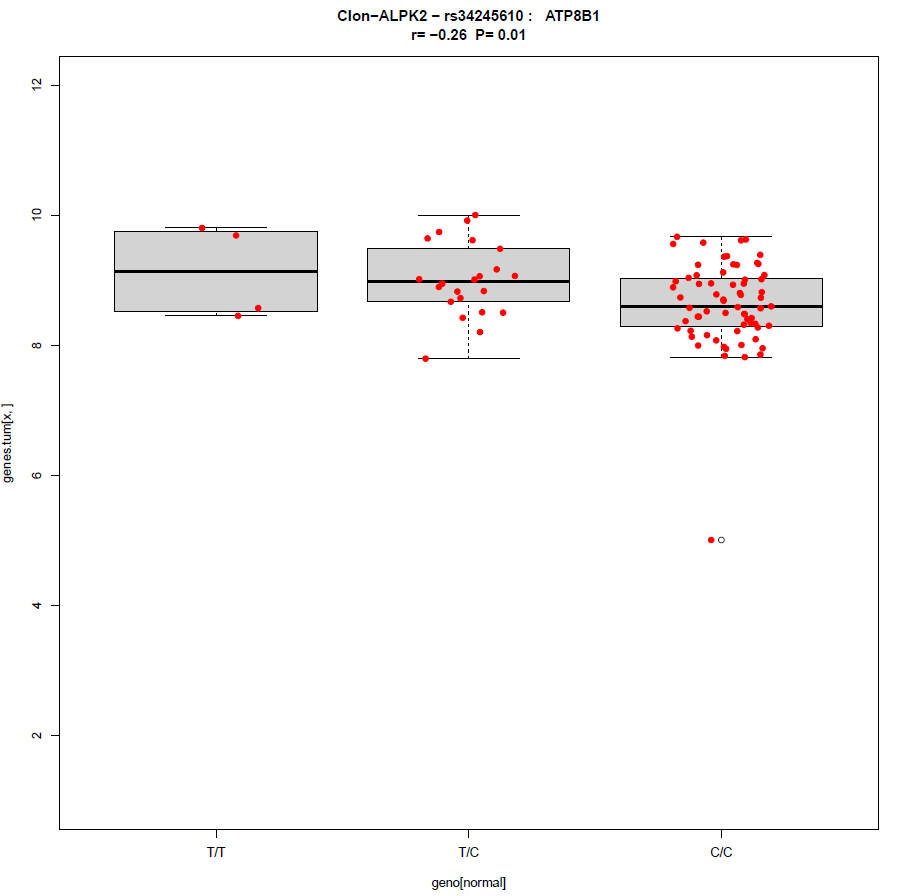


Figure S10. Q-Q plot for abundance from Discovery GWAS (N_snp_=9,636,751).


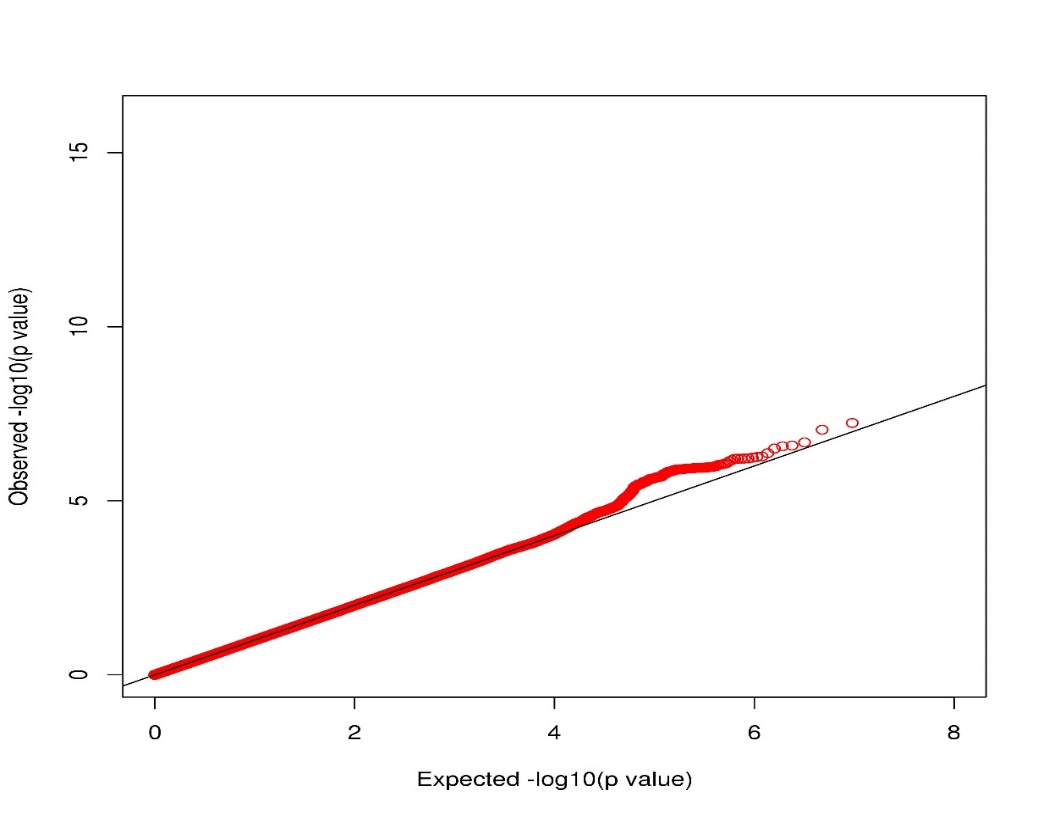


Figure S11. Manhattan plot for abundance from Discovery GWAS (N_snp_=9,636,751).


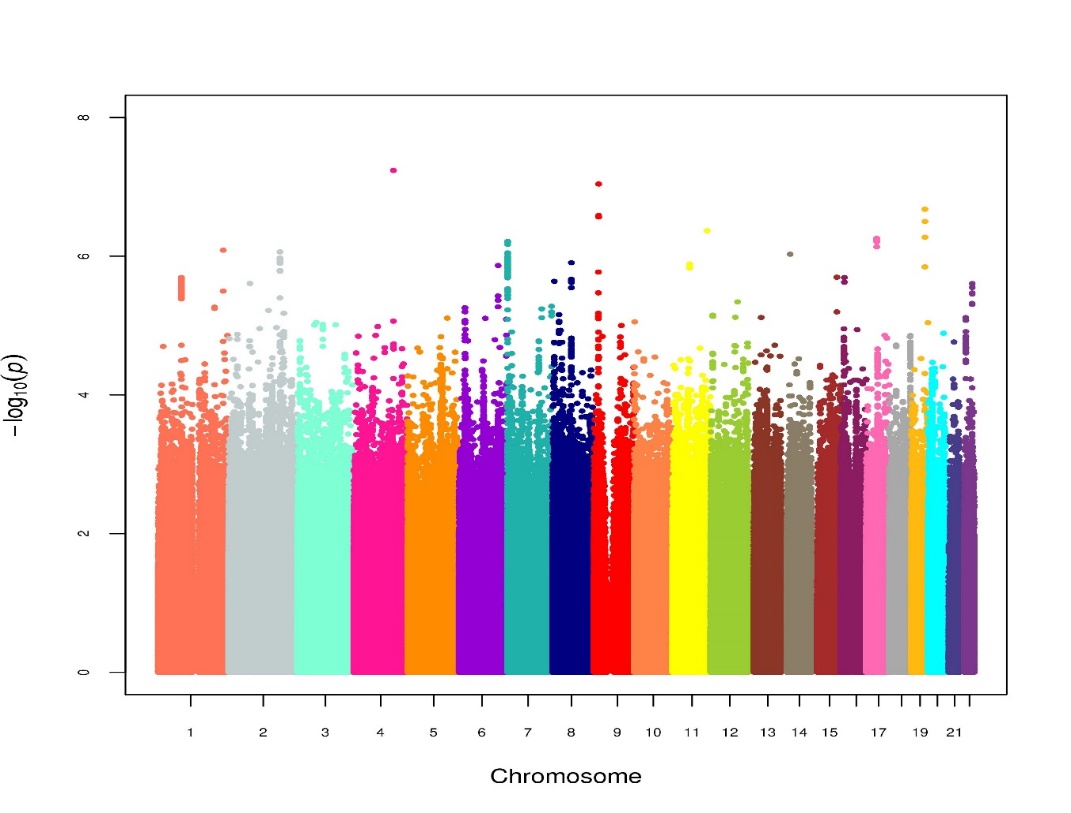


*Figure S12. eQTL result for rs56148061 with SYT7 using Colonomics colon tumor tissue transcriptomic data.*


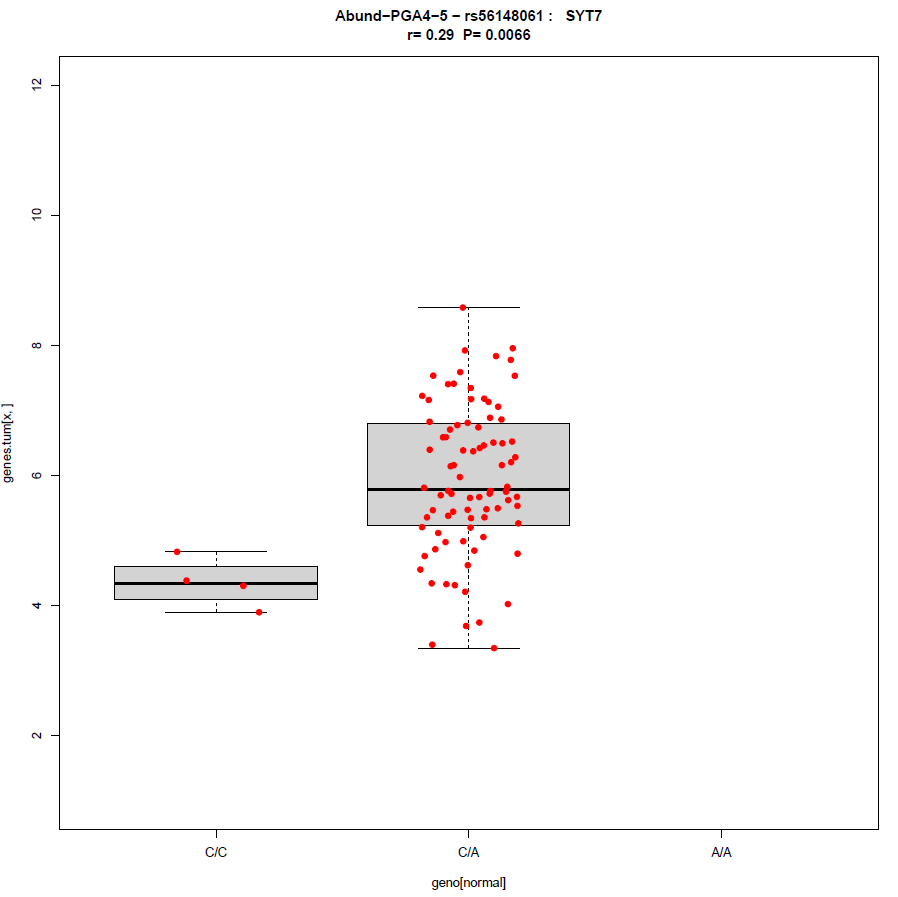


*Figure S13. Q-Q plot for TILs/hpf from Discovery GWAS (N_snp_=9,529,506).*


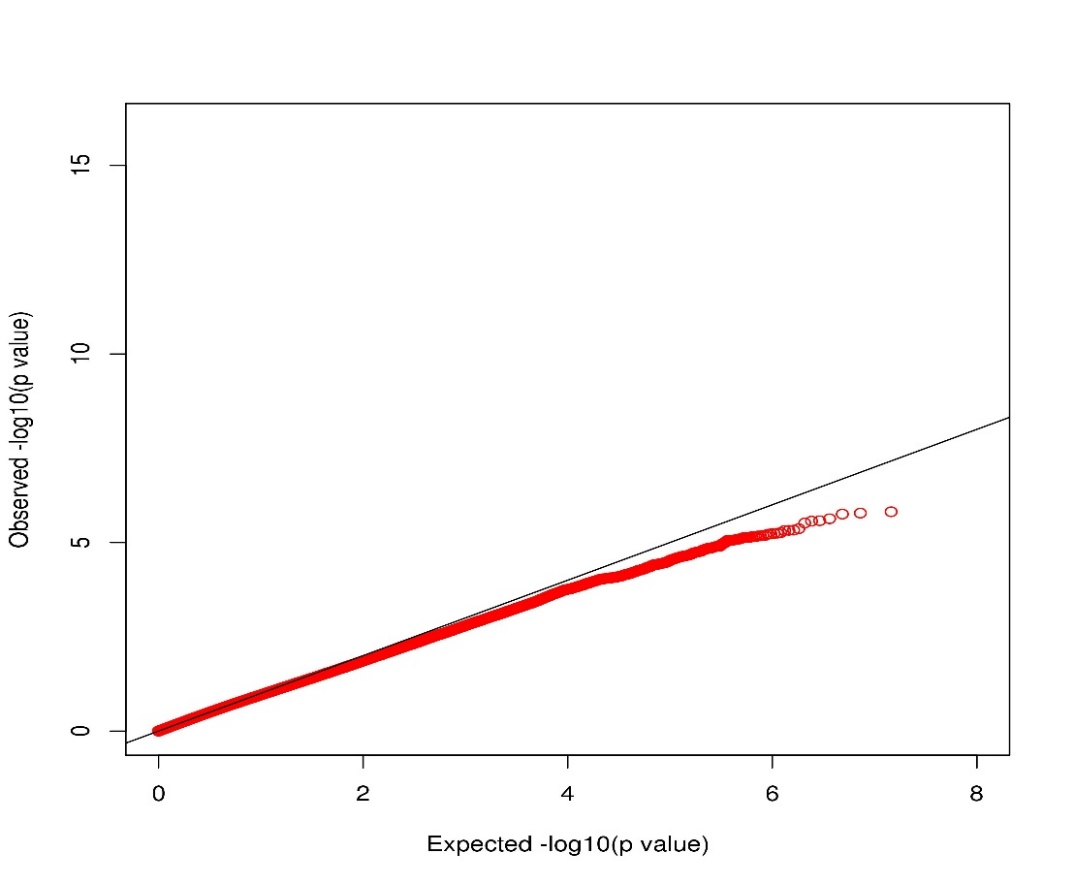


Figure S14. Manhattan plot for TILs/hpf from Discovery GWAS (N_snp_=9,529,506).


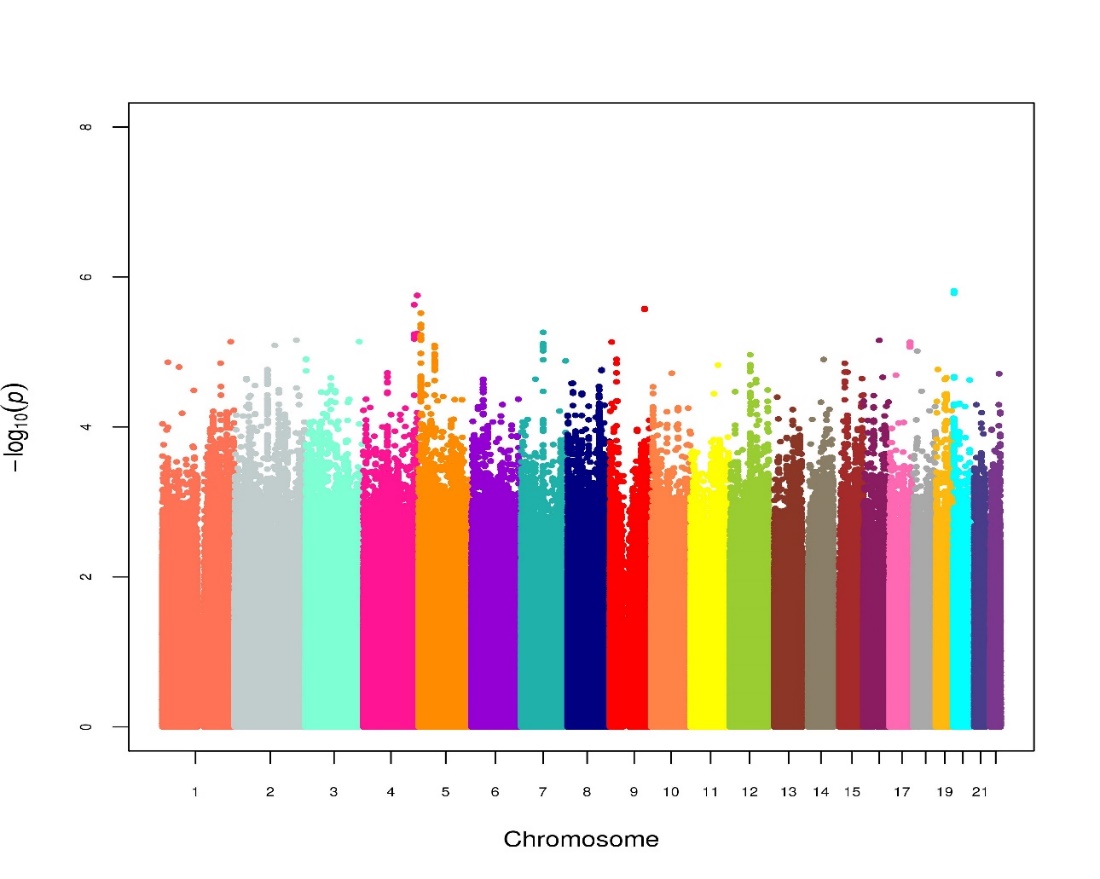


Figure S15. eQTL results for rs10982853 with TNFSF8 using BarcUVa-Seq healthy colonic mucosa transcriptomic data.

P=0.0139

*Figure S16. eQTL result for rs10982853 with PAPPA using Colonomics colon tumor tissue transcriptomic data.*

*
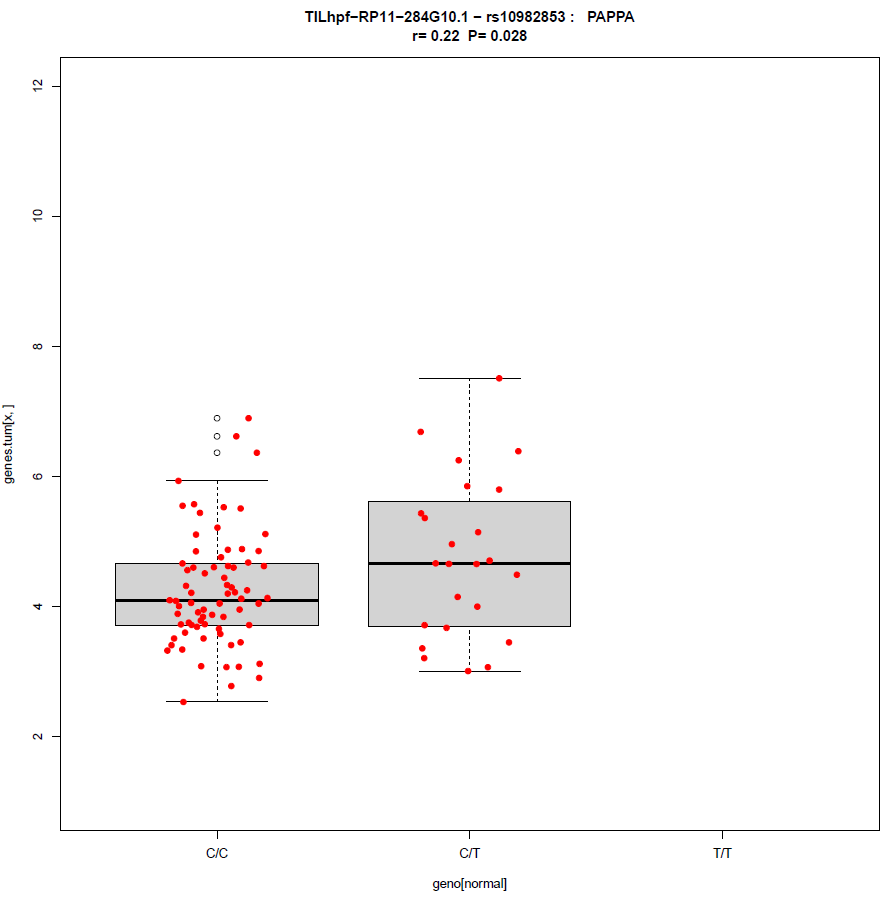
*

Figure S17. eQTL results for rs215529 with FASTKD5 and CPXM1 using BarcUVa-Seq healthy colonic mucosa transcriptomic data.

P=0.0369

P=0.0277

*Figure S18. eQTL result for rs215529 with PDYN using Colonomics colon tumor tissue transcriptomic data.*


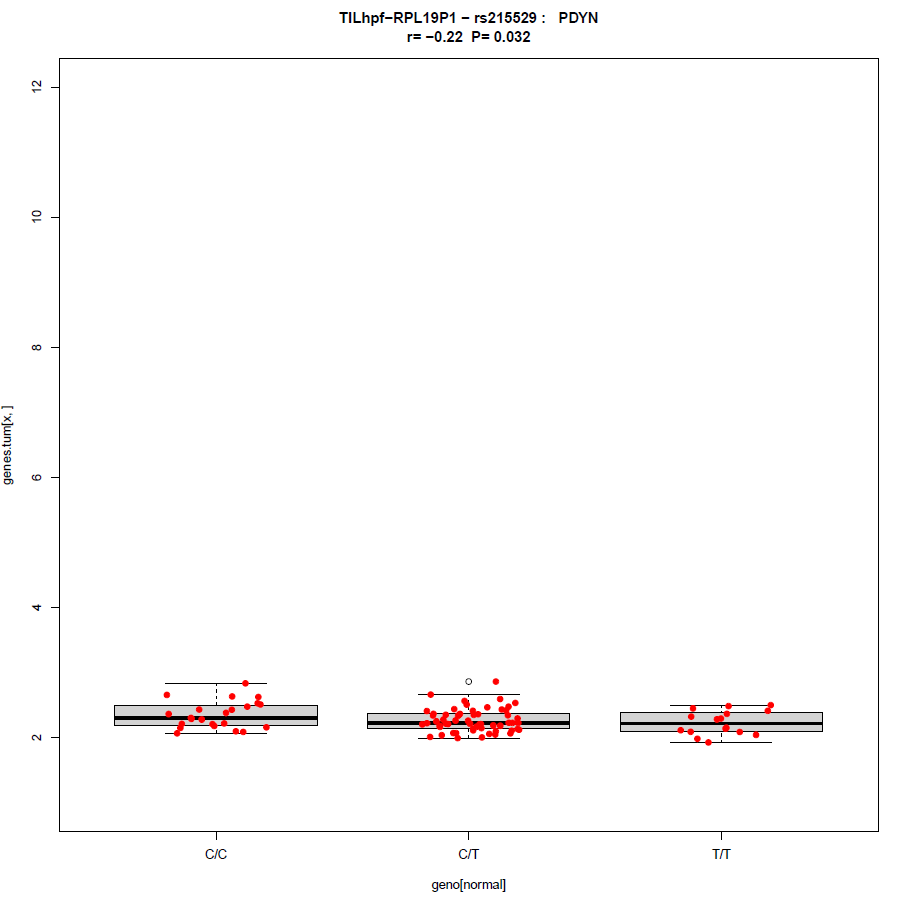


*Figure S19. Violin plots for the top SNPs associated with clonality and abundance.*

| 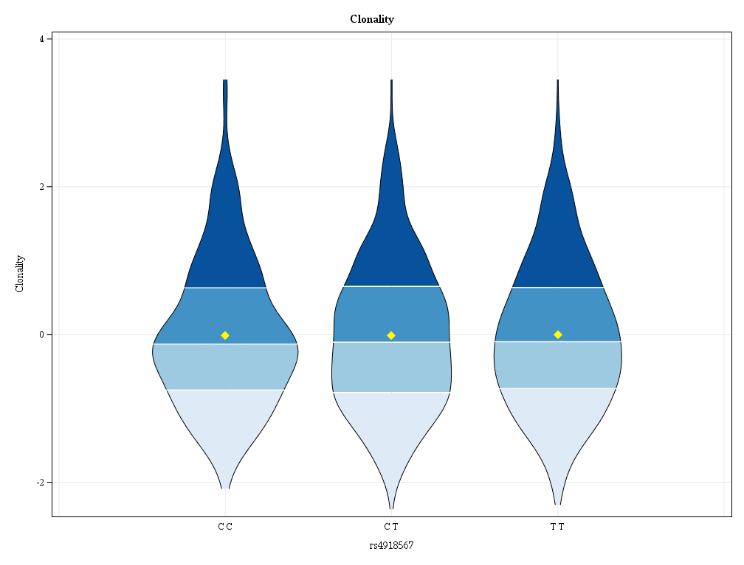 | 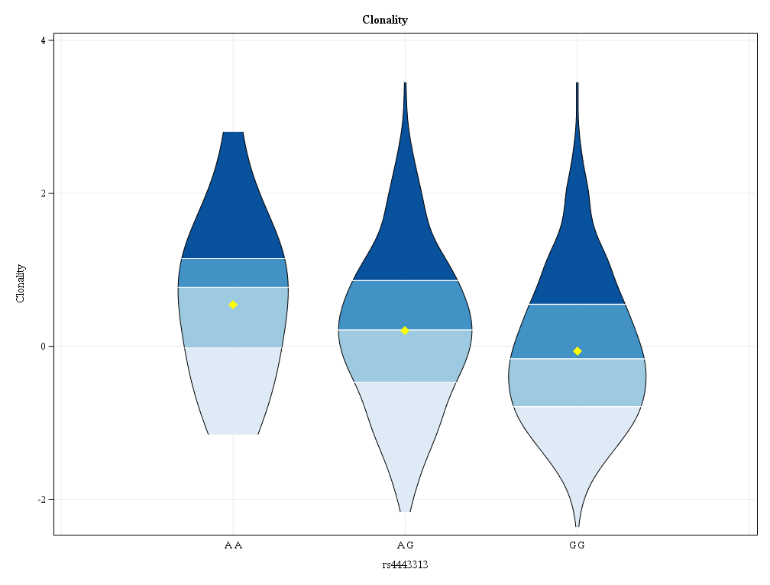 |
| --- | --- |
| 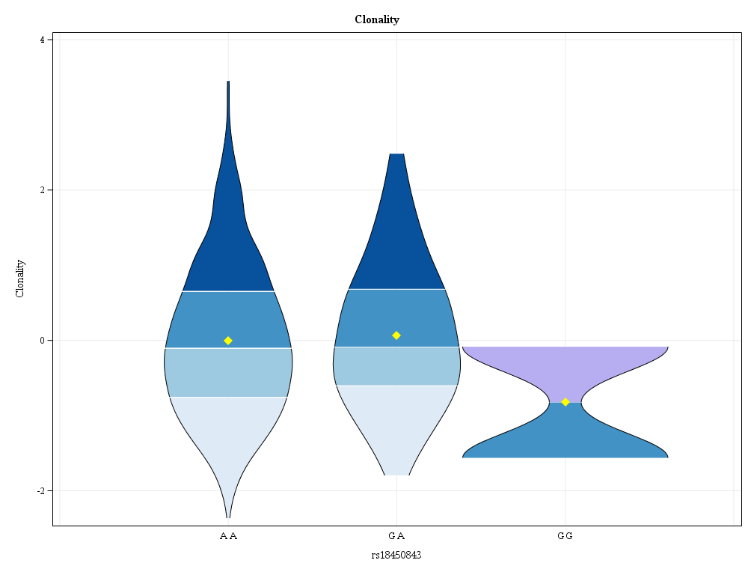 | 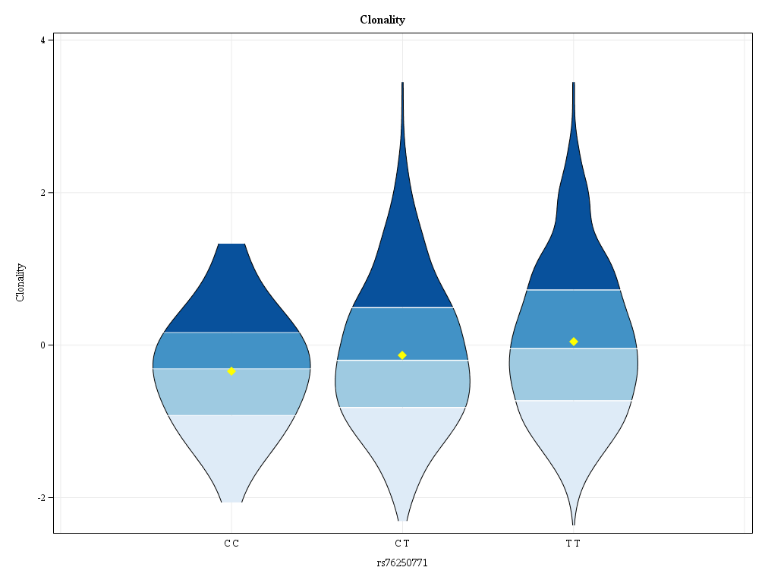 |
| 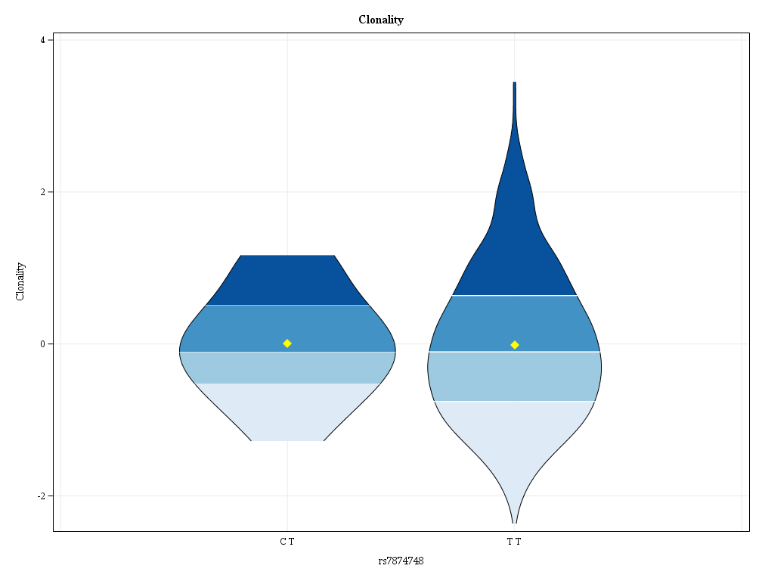 | 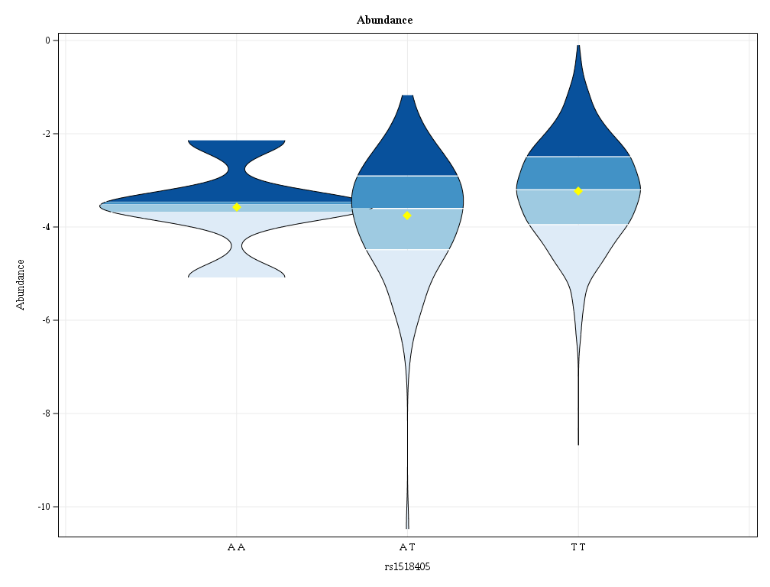 |
| 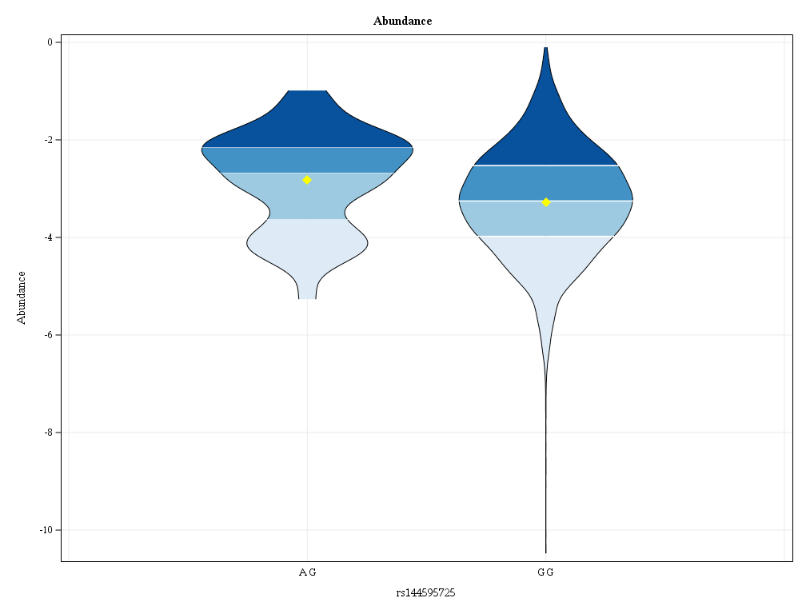 | 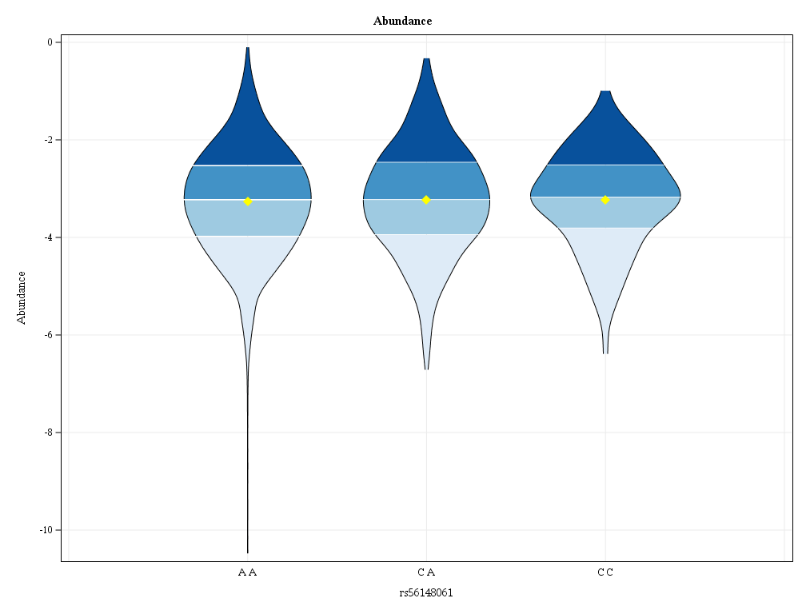 |
| 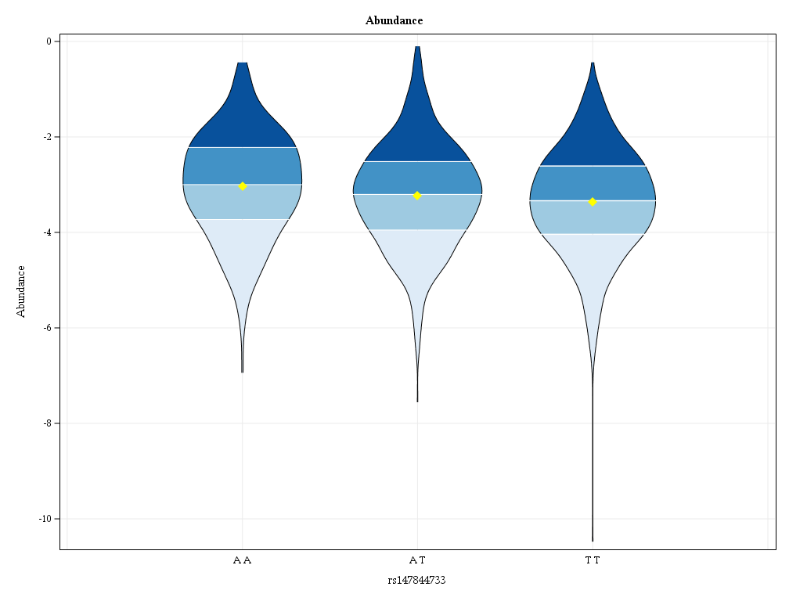 | 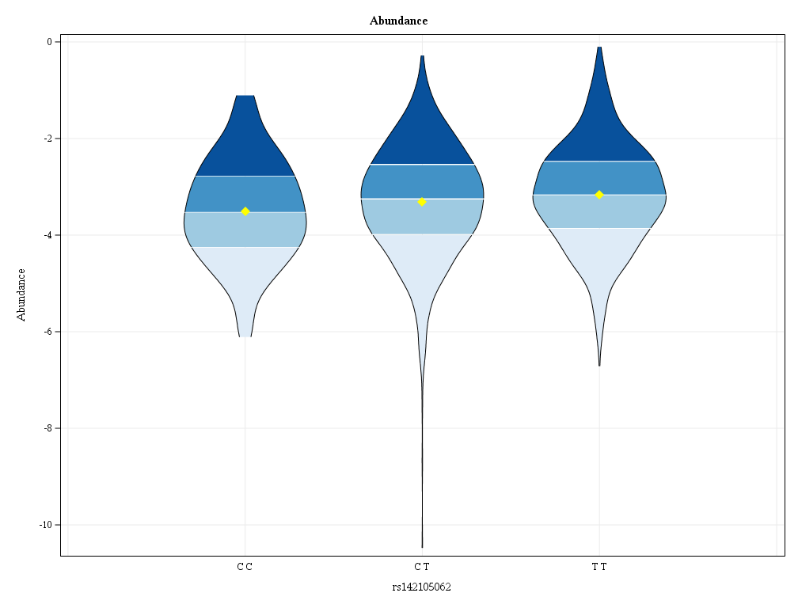 |
| 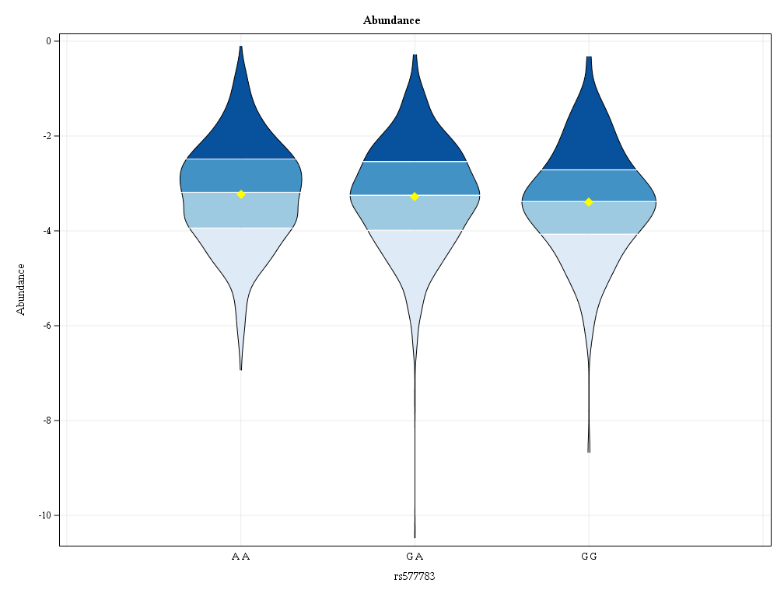 | 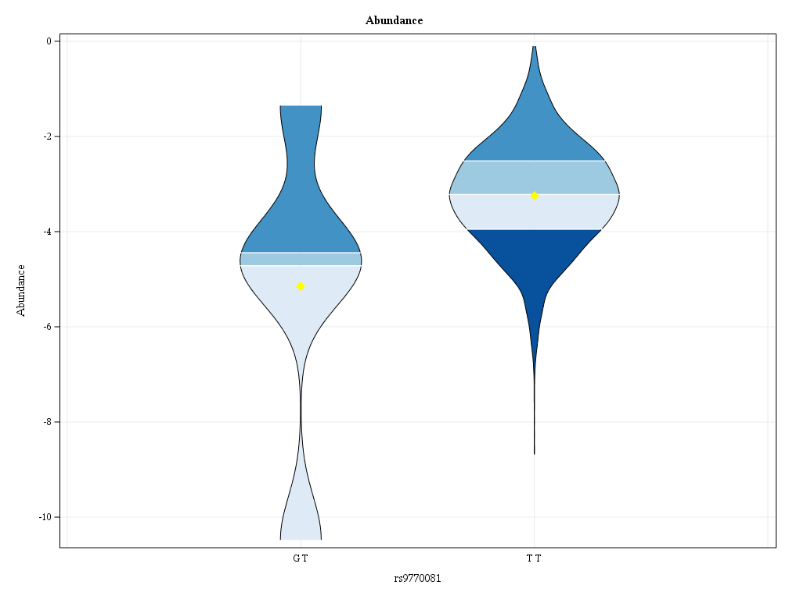 |
| 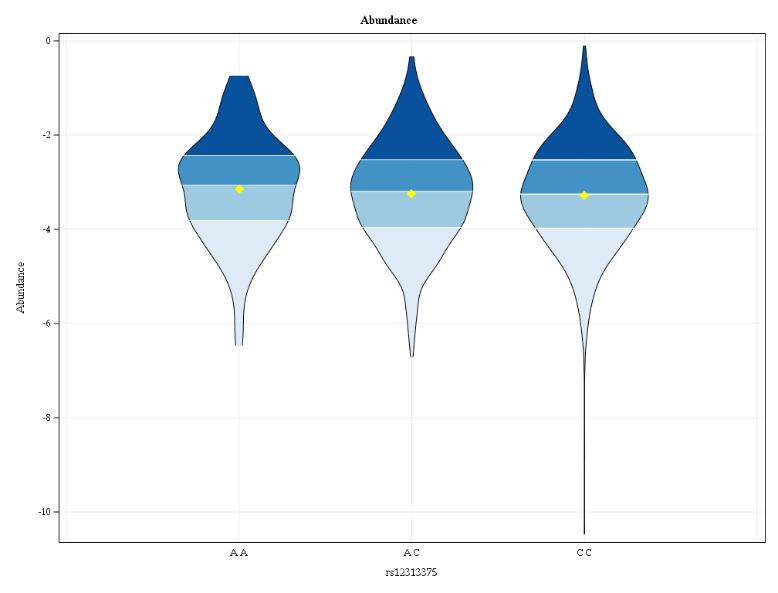 | 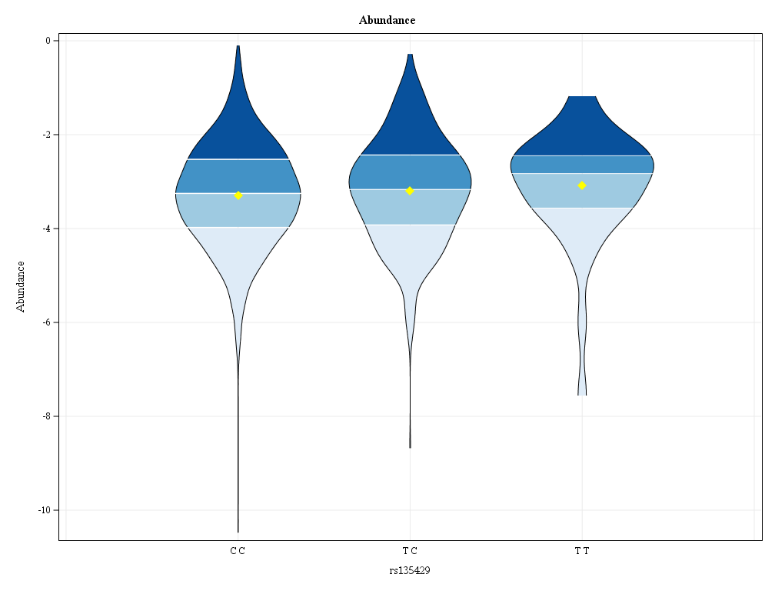 |
| 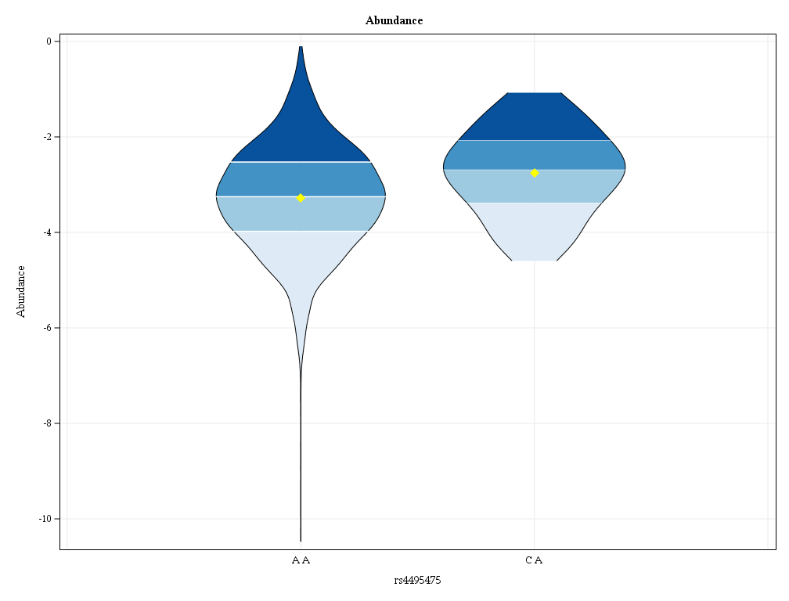 | 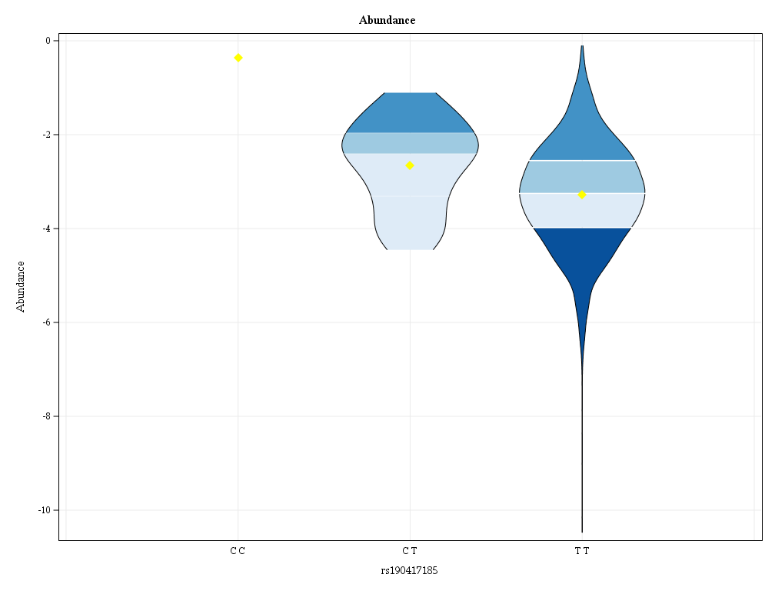 |
